# Modulated TRPC1 Expression Predicts Sensitivity of Breast Cancer to Doxorubicin and Magnetic Field Therapy: Segue Towards a Precision Medicine Approach

**DOI:** 10.3389/fonc.2021.783803

**Published:** 2022-01-24

**Authors:** Yee Kit Tai, Karen Ka Wing Chan, Charlene Hui Hua Fong, Sharanya Ramanan, Jasmine Lye Yee Yap, Jocelyn Naixin Yin, Yun Sheng Yip, Wei Ren Tan, Angele Pei Fern Koh, Nguan Soon Tan, Ching Wan Chan, Ruby Yun Ju Huang, Jing Ze Li, Jürg Fröhlich, Alfredo Franco-Obregón

**Affiliations:** ^1^ Department of Surgery, Yong Loo Lin School of Medicine, National University of Singapore, Singapore, Singapore; ^2^ Biolonic Currents Electromagnetic Pulsing Systems Laboratory (BICEPS), National University of Singapore, Singapore, Singapore; ^3^ Lee Kong Chian School of Medicine, Nanyang Technological University Singapore, Singapore, Singapore; ^4^ Cancer Science Institute of Singapore, National University of Singapore, Singapore, Singapore; ^5^ School of Biological Sciences, Nanyang Technological University Singapore, Singapore, Singapore; ^6^ Division of General Surgery (Breast Surgery), Department of Surgery, National University Hospital, Singapore, Singapore; ^7^ Division of Surgical Oncology, National University Cancer Institute, Singapore, Singapore; ^8^ Department of Obstetrics & Gynaecology, Yong Loo Lin School of Medicine, National University of Singapore, Singapore, Singapore; ^9^ Fields at Work GmbH, Zürich, Switzerland; ^10^ Institute of Electromagnetic Fields , ETH Zürich (Swiss Federal Institute of Technology in Zürich), Zürich, Switzerland; ^11^ Institute for Health Innovation & Technology (iHealthtech), National University of Singapore, Singapore, Singapore; ^12^ Competence Center for Applied Biotechnology and Molecular Medicine, University of Zürich, Zürich, Switzerland; ^13^ Department of Physiology, Yong Loo Lin School of Medicine, National University of Singapore, Singapore, Singapore; ^14^ Healthy Longevity Translational Research Programme, Yong Loo Lin School of Medicine, National University of Singapore, Singapore, Singapore

**Keywords:** breast cancer, PEMFs, EMT, patient-derived xenograft, chorioallantoic membrane, doxorubicin, TRPC1, chemotherapy

## Abstract

Chemotherapy is the mainstream treatment modality for invasive breast cancer. Unfortunately, chemotherapy-associated adverse events can result in early termination of treatment. Paradoxical effects of chemotherapy are also sometimes observed, whereby prolonged exposure to high doses of chemotherapeutic agents results in malignant states resistant to chemotherapy. In this study, potential synergism between doxorubicin (DOX) and pulsed electromagnetic field (PEMF) therapy was investigated in: 1) MCF-7 and MDA-MB-231 cells *in vitro*; 2) MCF-7 tumors implanted onto a chicken chorioallantoic membrane (CAM) and; 3) human patient-derived and MCF-7 and MDA-MB-231 breast cancer xenografts implanted into NOD-SCID gamma (NSG) mice. *In vivo*, synergism was observed in patient-derived and breast cancer cell line xenograft mouse models, wherein PEMF exposure and DOX administration individually reduced tumor size and increased apoptosis and could be augmented by combined treatments. In the CAM xenograft model, DOX and PEMF exposure also synergistically reduced tumor size as well as reduced Transient Receptor Potential Canonical 1 (TRPC1) channel expression. *In vitro*, PEMF exposure alone impaired the survival of MCF-7 and MDA-MB-231 cells, but not that of non-malignant MCF10A breast cells; the selective vulnerability of breast cancer cells to PEMF exposure was corroborated in human tumor biopsy samples. Stable overexpression of TRPC1 enhanced the vulnerability of MCF-7 cells to both DOX and PEMF exposure and promoted proliferation, whereas TRPC1 genetic silencing reduced sensitivity to both DOX and PEMF treatments and mitigated proliferation. Chronic exposure to DOX depressed TRPC1 expression, proliferation, and responses to both PEMF exposure and DOX in a manner that was reversible upon removal of DOX. TRPC1 channel overexpression and silencing positively correlated with markers of epithelial-mesenchymal transition (EMT), including *SLUG*, *SNAIL*, *VIMENTIN*, and *E-CADHERIN*, indicating increased and decreased EMT, respectively. Finally, PEMF exposure was shown to attenuate the invasiveness of MCF-7 cells in correlation with TRPC1 expression. We thus demonstrate that the expression levels of TRPC1 consistently predicted breast cancer sensitivity to DOX and PEMF interventions and positively correlated to EMT status, providing an initial rationale for the use of PEMF-based therapies as an adjuvant to DOX chemotherapy for the treatment of breast cancers characterized by elevated TRPC1 expression levels.

## Introduction

Breast cancer is the leading cause of cancer-associated death for women worldwide (1). Approximately 1 in 8 women in the US will be diagnosed with invasive breast cancer within their lifetimes (2) and, although chemotherapy is the mainstream treatment modality for breast cancer, greater than 50% of women undertaking chemotherapy will experience at least one chemotherapy-related adverse event (3). An urgent need exists for companion therapies to improve chemotherapeutic outcome in hopes of mitigating associated adverse events and reducing treatment-related toxicities.

Doxorubicin (DOX) is the most widely used chemotherapeutic agent for breast and other cancers (3). The anticancer effects of DOX are attributed to its ability to both inhibit DNA replication in actively-proliferating cancer cells (4) and to augment the production of reactive oxygen species (ROS) *via* disruption of redox cycling, thereby causing oxidative damage to lipids, DNA, and proteins (3). The ensuing mitochondrial damage further accentuates DOX-related ROS production to exacerbate oxidative damage (4).

Brief exposure (10 min) to low amplitude (1 mT) pulsing magnetic fields (PEMFs) has been shown capable of stimulating mitochondrial respiration and ROS production (5), thereby promoting both *in vitro* (5) and *in vivo* (6) myogeneses *via* a process of Magnetic Mitohormesis. Obeying a mitohormetic mechanism of operation (7), brief and low amplitude PEMF exposure would produce sufficiently low levels of ROS to instill mitochondrial survival adaptations, whereas exaggerated PEMF exposure might be expected to produce detrimental oxidative stress that would instead stymie cell survival. Importantly, the threshold for achieving an irreversibly damaging level of oxidative stress would depend on the basal metabolic rate and the existing inflammatory status of the recipient cells. Cancers characterized by elevated metabolic rates might hence be preferentially susceptible to PEMF-induced metabolic catastrophe (8, 9). Accordingly, exposure to 3 mT PEMFs for one hour was previously shown to be cytotoxic to MCF-7 breast cancer cells, whereas the same exposure paradigm was tolerated by MCF10A nonmalignant breast cells (10).

Transient Receptor Potential Canonical 1 (TRPC1) channel expression is necessary and sufficient to bestow PEMF-stimulated mitochondrial respiration (11). Evidence of a TRPC1-mitochondrial axis exists with the findings that calcium entry modulates mitochondrial respiration (12), whereas mitochondrial ROS reciprocally modulates TRPC1 function (13). TRPC1-mediated calcium was identified as an exploitable point of vulnerability to undermine cancer viability (14, 15) by commandeering the calcium/ROS-dependent cytotoxicity pathway (16, 17). TRPC1 and TRPM7 are the most abundantly expressed of all TRP channels (18), underscoring their physiological and hence, clinical importance. Elevated expression levels of TRPC1, TRPC6, TRPM7, TRPM8, and TRPV6 are detected in human breast ductal adenocarcinoma (hBDA) cells (19), whereby the expressions of TRPC1, TRPM7, and TRPM8 were most closely correlated with proliferative deregulation and tumor growth, and TRPV6 was more strongly correlated in invasive breast cancers. On the other hand, in high histopathological grade ovarian cancer, TRPC1 expression was negatively correlated with chemoresistance (20). Conversely, DOX treatment has been shown to induce genotypic and phenotypic modifications that make cancer cells refractory to chemotherapy (21). While the chemotherapeutic agents, cisplatin and carboplatin, are capable of downregulating TRPC1 channel expression in ovarian cancer cell lines (20), the effect of DOX on TRPC1 channel expression in breast cancer is unexplored.

Given the reported capacity of PEMFs to target breast cancer cells as well as TRPC1 (5, 10), we hypothesized that the effects of DOX and PEMF treatments might synergize to undermine breast cancer growth. We examined the independent and combined potentials of DOX and PEMF treatments both *in vitro* and *in vivo* to undermine cancer growth. Furthermore, given previous evidence demonstrating that PEMF exposure modulates TRPC1 function (5), we examined whether TRPC1 expression level predicts breast cancer vulnerability to DOX and PEMF treatments. Overexpression and silencing experiments were conducted to confirm detected trends in PEMF and DOX sensitivities related to endogenous TRPC1 channel expression as well as to investigate the propensity of the resultant breast cancer cells to undergo epithelial-mesenchymal transition (EMT) that would provide clinical relevance to the heightened sensitivities to PEMFs and DOX conferred by TRPC1.

## Materials and Methods

### MCF-7 Breast Cancer Xenograft and Patient-Derived Xenograft (PDX) Model in NSG Mice

NSG (NOD.Cg-Prkdc^scid^ Il2rg^tm1Wjl^/SzJ) mice, which lack human-specific cytokines and human leukocyte antigen (HLA) expression on stromal cells were used to host the breast cancer cell lines or patient-derived xenografts (PDX) (22). The NSG mice were purchased from The Jackson’s Laboratory and used at 8-10 weeks of age. Briefly, each female NSG mouse was implanted with a subcutaneous 60-day (0.36 mg) slow-release estradiol pellet (Innovative Research of America). Each patient tumor was equally divided into 5 chunks and implanted into the dorsal flank of 5 animals corresponding to the 5 different treatment groups. The tumors were allowed to grow for 3 weeks. For MCF-7, 1x10^6^ cells were counted and mixed in a 40-60% ratio with Matrigel Growth Factor (Bio Laboratories, Cat No. 354230). The cells are injected subcutaneously into the dorsal flank of the animals corresponding to the different treatment groups. The animals were given 20 mg/kg DOX intravenously and/or PEMFs stimulation for 1 h weekly for 5 weeks. At the end of the study, tumor volume was measured and isolated for apoptotic cell determination.

### Chick Chorioallantoic Membrane (CAM) Model

The chick chorioallantoic membrane (CAM) assay (23) was performed using fertilized Bovans Goldline Brown chicken eggs purchased from Chew’s Agriculture Pte Ltd and Lian Wah Hang Farm Pte Ltd, Singapore. Briefly, eggs were placed horizontally in a 38.5°C humified chamber of 70% humidity for 3 days. On day 3, 3 to 4 ml of albumin was removed through a hole in the apex of the eggs using an 18G needle on a 5 ml syringe to lower the CAM. An oval 1 cm^2^ hole was then made on the center of the eggs and covered using a 1624W Tegaderm semi-permeable membrane. On day 7, the eggs were inoculated with 1.5 x 10^6^ MCF-7 cells resuspended in 50 µl of Matrigel (Sigma Aldrich) on the blood vessel of the CAM. Prior to the inoculation of the MCF-7 cells, the blood vessels closer to the CAM were gently perforated using a dry glass rod. The eggs were resealed using Tegaderm and left for another 3 days. The eggs were then exposed to PEMF stimulation on days 10, 11, 12 and 13 for 1 h each day. Tumor weight was determined on Day 14 and subsequently processed for Western analysis. For the administration of DOX, DOX in saline at a concentration of 0.04 µg per gram of egg was prepared in a total volume of 20 µl, and added onto a small sterile filter paper placed on the CAM vessel next to the tumor. DOX was added 1 h prior to the first PEMF exposure.

### Histological Analysis and TUNEL Assay

Isolated MCF-7 tumors and chicken embryo livers were fixed in 2.5% PFA and 15% sucrose in PBS for 24 h at 4°C. Tissues were embedded in cryoprotectant Tissue-Tek^®^ O.C.T. Compound and were sectioned at 10 µM thickness. TUNEL assay was performed using Click-iT Plus TUNEL Assay kit (Thermo Fisher Scientific) according to manufacturer’s protocol. For human breast biopsies, the tissues were kept in RPMI media supplemented with 10% FBS and exposed to 3 mT PEMF for 1 h. They were maintained in a standard tissue culture incubator for 24 h before fixation using 4% PFA overnight, which were subsequently processed and embedded in paraffin blocks. Tissue biopsies were sectioned at 5 µm thickness and stained with *In Situ* Cell Death Detection Kit (Roche) as per manufacturer’s instruction. The stained sections were viewed using Olympus FV1000 fluorescence microscope.

### Cell Culture and Pharmacology

MCF-7 (HTB-22™) cells were acquired from American Type Culture Collection (ATCC) and maintained in RPMI (Gibco) supplemented with 10% FBS (Hyclone) and maintained in a humidified incubator at 37°C in 5% CO_2_. MDA-MB-231 cells were a kind gift from Dr. Glenn Bonney, NUS and were authenticated by ATCC using human STR (short tandem repeat) profiling. MCF10A cells were acquired from Dr. Andrew Tan’s laboratory (NTU). MDA-MB-231 cells were maintained in DMEM (Gibco) and 10% FBS. MCF10A cells were maintained in growth media containing DMEM/F12 (Gibco) supplemented with 5% horse serum (Hyclone), 20 ng/ml EGF (Peprotech), 0.5 mg/ml hydrocortisone (Sigma), 100 ng/ml cholera toxin (Sigma) and 10 ,µg/ml insulin (Sigma). Cells were trypsinized and passaged every 3 days using TrypLE Express reagent (Gibco). MCF-7/ADR cells resistant to 96 nM DOX were generated using a progressive incubation of cells in low 0.3 nM up to 96 nM DOX over 4 months. The concentration of DOX was doubled weekly upon cell reseeding. Doxorubicin hydrochloride (DOX) (Abcam, ab120629) was reconstituted in DMSO to make a stock concentration of 25 mM and stored at -80°C. Subsequent dilutions of DOX were made in distilled water to keep DMSO concentration below 0.01%. No cell culture antibiotics were used throughout the experiments.

### Cell Count and DNA Content Analysis

For cell enumeration using trypan blue exclusion assay, MCF-7, MDA-MB-231, or MCF10A cells were seeded at 6000 cells/cm^2^ per well of a 6-well plate. For MCF10A cells, they were plated in growth media without EGF. Cell counting was performed using 3 wells of a 6-well plate for technical replication. For DNA content analysis using Cyquant cell proliferation assay (Invitrogen), cells were seeded at 2000 cells per well and performed with 8 technical replicates in a 96-well plate. Seeded cells were left for 24 h before treatment with DOX or exposed to PEMFs. Cyquant stained DNA was measured using at 480/520 nm using Cytation 5 microplate reader (BioTek).

### Clonogenic Assay and Quantification of Colonies

*In vitro* clonogenic assay was performed using crystal violet staining (24). Briefly, MCF-7 cells were seeded either at 100 or 1000 cells per well of a 6-well plate. The cells were treated with DOX on Day 1, 4, and 7 in RPMI supplemented with 10% FBS. 3 mT PEMFs stimulation was administered for 1 h from Day 1 to Day 10. On Day 11, the cells were rinsed in PBS and stained with crystal violet stain consisting of 0.5% crystal violet and 6% glutaraldehyde (Sigma Aldrich) in distilled water for 3 h. Stained colonies were rinsed with 2 changes of tap water and left to dry. Images of the colonies were taken using Chemidoc Imaging System (Bio-Rad) under the Coomassie Blue Stain filter setting. The number of colonies and colony size per well was estimated using the ImageJ Analyze particle option using 3 to 3500-pixel unit with a circularity of 0.2 to 1. The mean survival factor (colony count) was determined as the number of surviving cells over the number of cells plated and normalized to the survival factor of the control group expressed as fold change. The colony size relative frequency was determined by binning colonies into several bins, according to their relative size from the smallest to the largest colonies after normalizing to the total number of cells.

### Reactive Oxygen Species Analysis Using DCH_2_FDA

MCF-7 cells were seeded into 96-well all black well plates (Costar) at a density of 5,000 cells per well with 7 replicates per condition and left to settle for 24 h. Cells were then treated with DOX at concentrations ranging between 20 nM and 50 µM in RPMI media supplemented with 10% FBS for 16-18 h. The cells were rinsed with warm phenol-free and serum-free (PFSF) RPMI (Gibco) and incubated with 10 µM DCH_2_FDA (Invitrogen) in PFSF RPMI. The cells were then exposed to 0 mT or 3 mT PEMFs for 30 min, followed by washing out of the remaining extracellular dye with warm PFSF RPMI and left in the standard incubator for 30 min before proceeding to ROS determination using a Cytation 5 microplate reader (BioTek) at Ex/EM: 492/520 nm every hour.

### Intracellular Calcium Determination

MCF-7 cells were seeded into 96-well clear bottom black well plates (Costar) at a density of 5,000 cells per well with 7 replicates per condition and left to settle for 24 h. Cells were treated with DOX at concentrations ranging between 20 nM and 50 µM in RPMI media supplemented with 10% FBS for 16-18 h. Cells were then rinsed with room temperature phenol-free and serum-free (PFSF) RPMI (Gibco) and incubated with 1 µM Calcium Green-1 AM (Invitrogen) in PFSF RPMI. The cells were then immediately exposed to 0 mT or 3 mT PEMFs for 30 min at room temperature, followed by washing away of the excess extracellular dye using PFSF RPMI before proceeding to calcium fluorescence measurement using a Cytation 5 microplate reader (BioTek) at Ex/EM: 506/531 nm after 25 min incubation at room temperature.

### Western Analysis

Cell lysates were prepared in ice-cold radioimmunoprecipitation assay (RIPA) buffer containing 150 mM NaCl, 1% Triton X-100, 0.5% sodium deoxycholate, 0.1% SDS and 50 mM Tris (pH 8.0) supplemented with protease (Nacalai Tesque) and phosphatase inhibitors (PhosphoSTOP, Roche). Cells were lysed for 20 min and centrifuged for 10 min at 13,500 rpm. The protein concentration of the soluble fractions was determined using a BCA reagent (Thermo Fisher Scientific). 25 - 50 µg of total protein was resolved using 10% or 12% denaturing polyacrylamide gel electrophoresis and transferred to PVDF membrane (Immobilon-P, PVDF). Proteins on PVDF membranes were blocked using 5% low-fat milk in TBST containing 0.1% Tween-20 and incubated with the primary antibody in SuperBlock TBS (Thermo Fisher Scientific) overnight at 4°C. The primary antibodies used were: TRPC1 (1:300; Santa Cruz), Cyclin D1 (CD1, 1:300; Santa Cruz), GFP (1:1000; Proteintech), β-actin (1:10,000; Proteintech), α-tubulin (1:5000; Proteintech). The membranes were washed in TBST. Anti-rabbit or anti-mouse antibody conjugated to horseradish peroxidase (HRP) were diluted (1:3000, Thermo Fisher Scientific) in 5% milk in TBST and were incubated with the membranes for 1 h at room temperature. The membranes were incubated in SuperSignal West Pico or West Femto chemiluminescent substrate (Thermo Fisher Scientific), detected and analyzed using LI-COR Image Studio.

### Laser Confocal Imaging

For the visualization of GFP and Vimentin abundance in TRPC1 overexpressing MCF-7 cells, the cells were seeded onto coverslips at a density of 100,000 cells per well of a 6-well plate (Nunc). 24 h post-seeding, the cells were rinsed with PBS and fixed in 4% paraformaldehyde for 20 min. For the direct visualization of the expression of GFP in vector-only and MCF-7/TRPC1 cells, the cells on coverslips were mounted onto glass slides using ProLong Gold Antifade Mountant (Thermo Fisher Scientific). The cells were then analyzed using the Olympus FV1000 confocal laser scanning microscope. For the visualization of Vimentin, the cells were permeabilized with 0.1% Triton in PBS for 10 min after fixation. The cells were then blocked in SuperBlock TBS (Thermo Fisher Scientific) followed by Vimentin antibody (Santa Cruz, 1:100) incubation overnight, followed by secondary Alexa Fluor 594 antibody (1:500, Thermo Fisher Scientific) for 1 h at room temperature. Washes between steps were done with PBS with 0.1% Tween (Sigma Aldrich). Nuclei of cells were co-stained with DAPI (Sigma Aldrich) for 10 min. Cells were finally mounted and visualized using a laser scanning confocal microscope. For the quantitative analysis of Vimentin abundance, the total absolute intensity per view was normalized to the number of nuclei to yield a mean protein intensity per cell. The average of the mean protein intensity per cell (at least 10 cells per view) from multiple replicates were used to compute and compare the abundance of Vimentin protein between vector-only and MCF-7/TRPC1 cells.

### Real-Time qPCR and TRPC1 Silencing

Quantitative reverse-transcription polymerase chain reaction (RT-qPCR) was carried out using the SYBR green-based detection workflow. Briefly, total RNA was harvested from MCF-7 cells using Trizol reagent (Thermo Fisher Scientific) and 0.5 µg of RNA was reverse transcribed to cDNA using iScript cDNA Synthesis kit (Bio-Rad). Quantification of gene transcript expression was performed using SSoAdvanced Universal SYBR Green (Bio-Rad) on the CFX Touch Real-Time PCR Detection System (Bio-Rad). Relative transcript expression was determined using the 2^-ΔΔCt^ method, normalized to β-actin transcript levels. The qPCR primers used were: *TRPC1*, F: 5’-AAG CTT TTC TTG CTG GCG TG, R: 5’-ATC TGC AGA CTG ACA ACC GT; *SNAIL*, F: 5’-CGA GTG GTT CTT CTG CGC TA, R: 5’-CTG CTG GAA GGT AAA CTC TGG A; *SLUG*, F: 5’-TAG AAC TCA CAC GGG GGA GAA G, R: 5’-ATT GCG TCA CTC AGT GTG CT; *VIMENTIN* F: 5’-AAG GCG AGG AGA GCA GGA TT, R: 5’- AGG TCA TCG TGA TGC TGA GA; and *β-ACTIN*, 5’-AGA AGA TGA CCC AGA TCA TGT TTG A, R: 5’-AGC ACA GCC TGG ATA GCA AC.

For TRPC1 silencing in MCF-7 cells, two pre-designed dicer-substrate short interfering RNAs (dsiRNA, IDT) were used to knock down the expression of TRPC1. Both dsiRNAs targeted the coding-sequence of TRPC1 (NM_001251845). Transfection of dsiRNA was performed using Lipofectamine 3000 reagent (Invitrogen) as per manufacturer’s protocol. TRPC1-silenced cells were validated using qPCR 48 h post dsiRNA transfection using primers against *TPRC1*, *SNAIL, SLUG* and *VIMENTIN* as indicated above, relative to cells transfected with scramble dsiRNA.

### Migration Assay

MCF-7 cells at a density of 30,000 cells in 120 µl RPMI supplemented with 10% FBS were seeded into each quadrant of a 4-well 3.5 mm culture dish insert (ibidi). The cells were left to adhere for 24 h before the removal of the insert and the addition of RPMI media containing 10% FBS to a total volume of 2 ml per dish. Closure of the gaps was captured using light microscopy on all four limbs of the insert, taken every 24 h. The average of 16 gap distances was considered from the 4 limbs with 4 readings arising from each limb. The images of the gap distances were analyzed using ImageJ.

### Invasion Assay

Invasion assay was performed using the CytoSelect 24-well Cell Invasion Assay kit (Cell Biolabs, Inc.) according to the manufacturer’s protocol. Briefly, 200,000 cells were seeded in the cell culture insert after the rehydration of the basal membrane in FBS-free RPMI media. The lower well of the invasion plate was filled with RPMI media supplemented with 10% to promote the invasion of cells through the basal membrane. 20 ng/ml TGFβ was added to selected conditions in the cell culture insert to stimulate cell invasion. The setup was incubated for 48 h in a standard tissue culture incubator before the extraction and staining of the invaded cells from the basal membrane. The lysates from the extracted cells were analyzed at OD 560 using Cytation 5 microplate reader (BioTek).

### Generation of Plasmid and Stable Cell Line

GFP-TRPC1 plasmid was generated by PCR amplification of full-length human TRPC1 cDNA (Accession: NM_001251845.2; 2382 base pairs) and directionally subcloned into the pEGFP-C1 vector. Transfection of plasmids in MCF-7 cells was carried out using Lipofectamine 3000 reagent (Invitrogen). 48 h after plasmid transfection, stable cells were selected in RPMI containing 750 µg/ml Geneticin (Invitrogen), 10% FBS, and 1% Pen/Strep (Gibco) in 5% CO_2_ at 37°C. GFP vector and GFP-TRPC1 cells were enriched for GFP positive cells using Beckman Coulter Moflo Astrios cell sorter. Stables cells were subsequently maintained in complete RPMI media containing 500 µg/ml Geneticin. The overexpression of GFP-TRPC1 in the stable cells was characterized using qPCR, immunofluorescence, and western analysis. GFP stable cells are referred to as vector-only cells while GFP-TRPC1 overexpression stables cells are referred to as MCF-7/TRPC1 cells in the manuscript.

### Apoptotic Assay

For apoptotic cell determination, the tumors were dissociated to single cells using the MACS Tumor Dissociation Kit in combination with the gentleMACS Dissociator (Miltenyi Biotec) as according to the manufacturer’s protocol. After dissociation, the cells were filtered through a 30 µm MACS SmartStrainer. Cells were pelleted from the filtrate at 300 g x 7 min and resuspended in 400 µl Binding Buffer. The cells were incubated with Annexin V FITC and Propidium Iodide (Sigma Aldrich) for 15 min in the dark at room temperature. After incubation, the cells were pelleted and resuspended in 100 µl Binding Buffer for analysis by flow cytometry using BD Accuri C6 cytometer (BD Biosciences, CA, USA).

### PEMF Exposure Paradigms

The principal PEMF device and magnetic signal used in this study has been previously described (10). The breast coil tested and debuted in this study generates the same signal as previously employed against breast cancer (10) and is based on a classical Helmholtz-coil configuration optimized for field uniformity within the dimensions of 120 mm height and 75 mm of radius (**Supplementary Figures 1A**–**C**). The coil dimensions were derived from existing clinical MRI breast scanning coils. The coil is accommodated in a standard patient bed to allow for comfortable positioning of the patients during the entire course of the exposure session (**Supplementary Figure 1D**).

The breast coil system was contract fabricated by Flex Ltd. (Singapore) in accordance with our specifications and consists of a field applicator module (field generating coil) and a power amplifier module. A proprietary coil configuration was unitized for optimal signal generation within the field applicator. A precision wire winding process ensured the generation of a uniform electromagnetic signal within the field applicator volume as previously described (10).

The amplifier module supports the power requirement for the field applicator to generate specified pulsed electromagnetic fields using a firmware fine-tuned with minimal heat dissipation, ensuring that the various signal output specifications are within defined tolerances. A power and current consumption safety monitoring module is designed to monitor current consumption of the field applicator in real-time with a feedback mechanism to the micro-controller. The system allows active interruption of treatment in the event of current over-flow or excessive field exposure to the subject using the device. The biological efficacy of the system was validated in a subset of the cell and animal experiments performed in this manuscript (**Supplementary Figures 1A**–**C**).

### Statistical Analysis

All statistics were carried out using GraphPad Prism (Version 9) software. Unless otherwise stated, statistical analyses were performed using One-Way analysis of variance (ANOVA) to compare the values between two or more groups followed by Bonferroni’s posthoc test. For the comparison between two independent samples, the Student’s *t*-test was performed.

## Results

### PEMF and Doxorubicin Treatments Act Synergistically to Impair Breast Cancer Tumor Growth *In Vivo*

**Figure 1:**
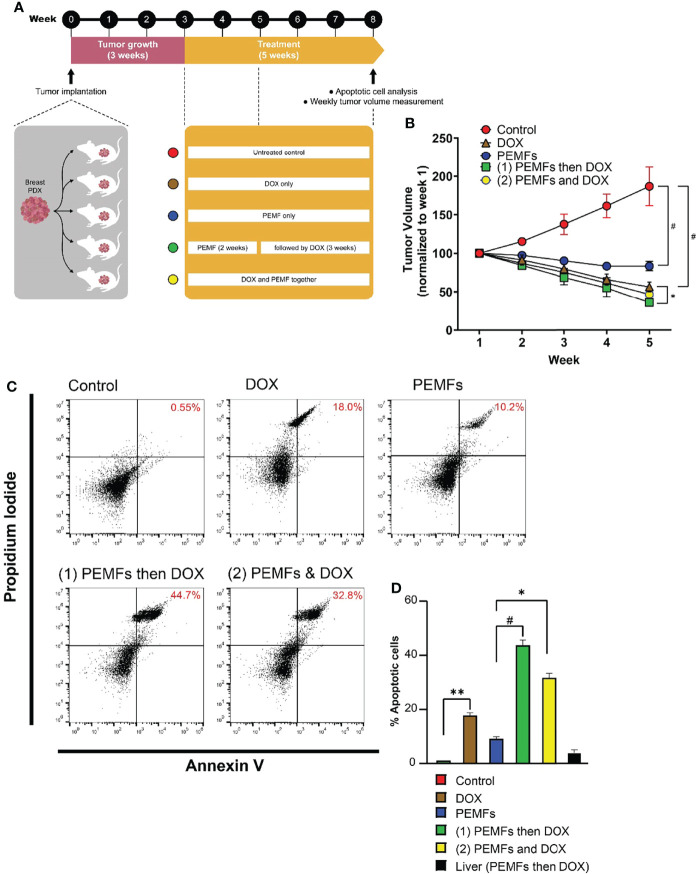
PEMFs synergize with DOX to inhibit tumor growth *in vivo*. **(A)** Schematic of PEMF and DOX exposure regimes used on mice hosting patient-derived tumor xenografts. Implanted tumors were allowed to grow for 3 weeks before the initiation of DOX (20 mg/kg) and/or PEMF treatments. Tumor volumes were measured each week while apoptotic cell determination was performed at the end of the study. Each data point represents the mean values from 5 experimental runs derived from the tumors obtained from 5 patients, each of which was equally divided amongst the 5 treatment groups. **(B)** Changes in tumor volume (mm^3^) for 5 weeks. **(C)** Representative scatter dot-plots showing cell populations from dissociated tumors based on Annexin V and propidium iodide staining. **(D)** Quantification of apoptotic cell percentages obtained using flow cytometry. *p < 0.05, **p < 0.01, and *^#^p* < 0.0001. Error bars represent the standard error of the mean.

PEMF exposure was previously shown to impair the viability of MCF-7 breast cancer cells when administered at an amplitude of 3 mT for 1 h per day (10). Here, we show that analogous PEMF exposure of immunocompromised NSG mice, hosting patient-derived breast tumor xenografts (PDX), showed a regression of breast tumor growth. Engrafted human tumors were allowed to grow in mice for 3 weeks prior to once-weekly exposure to 3 mT PEMFs for 1 h per week and/or intravenous administration of 20 mg/kg DOX. After 5 weeks tumors were harvested and analyzed (**Figure 1A**). Untreated (control) tumors showed a progressive increase in volume of ~90% from their initial values (**Figure 1B**; red). By contrast, PEMF treatment alone significantly reduced tumor volume by ~-20% (**Figure 1B**; blue) and doxorubicin (DOX) administration reduced tumor volumes by ~-50% (**Figure 1B**; brown) compared to starting values. Moreover, the incidence of apoptotic cells increased by +0.55%, +10.2%, and +18% in tumors isolated from control, PEMF-exposed and DOX-treated mice, respectively (**Figures 1C, D**). Synergism between PEMF and DOX interventions was revealed using two paradigms: 1) once weekly PEMF exposure for 2 weeks followed by 3 weeks of DOX treatment alone and; 2) simultaneous weekly PEMF and DOX treatments for 5 weeks. Amongst all the test conditions, paradigm 1 (**Figure 1B**, green) produced the greatest reductions in tumor volume (~-70%) and increases in apoptotic cells (~+45%) (**Figure 1D**, green), wherein tumor resorption (**Figure 1B**, green) was statistically different from DOX treatment alone (brown), but not from paradigm 2 (yellow). The livers of PEMF- then DOX-treated mice (paradigm 1) revealed no significant signs of collateral apoptosis (**Figure 1D**, black), demonstrating cytotoxic specificity for malignant tissues.

**Figure 2:**
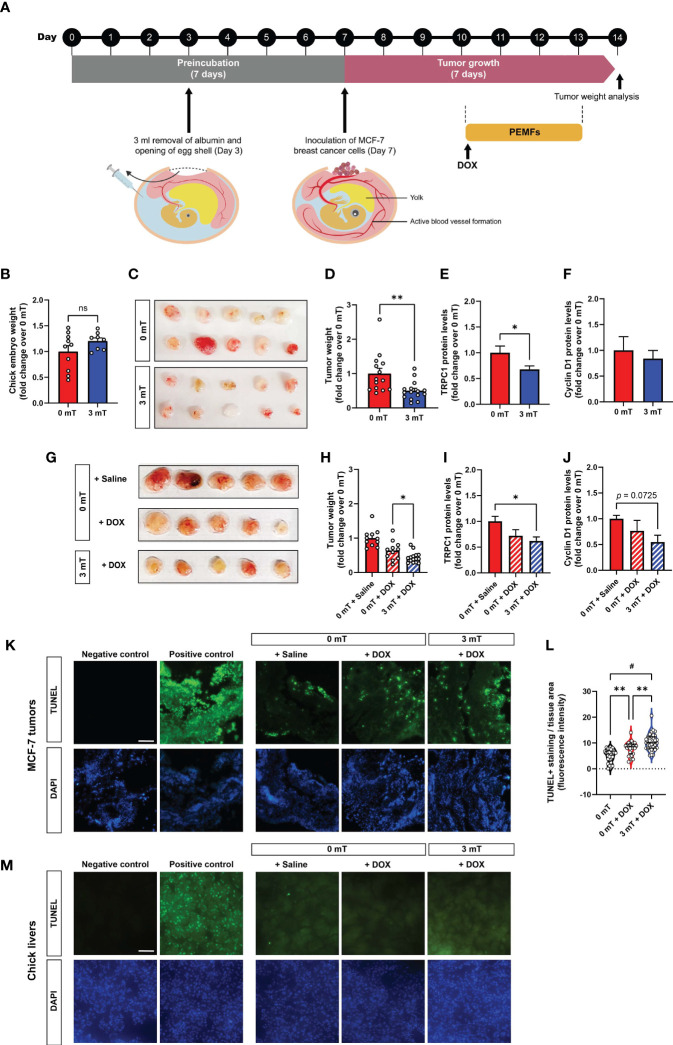
PEMF inhibits breast tumor growth *in vivo* correlated with depressed TRPC1 expression levels. **(A)** Schematic of the PEMF exposure paradigm used on the CAM model for MCF-7 breast tumor xenografts. MCF-7 tumors were inoculated onto the CAM on day 7. The tumors were either exposed to 3 mT for 1 h for 4 successive days or exposed to 3 mT after 1 h of DOX treatment (0.04 ug/g) from day 10 onwards. **(B)** Chick embryo weight at study termination on day 14 for both groups. **(C)** Representative images of MCF-7 tumor size at day 14 and corresponding quantification **(D)** represented as fold change over 0 mT. TRPC1 **(E)** and Cyclin D1 **(F)** expression levels normalized to GAPDH, respectively, expressed as fold change over 0 mT. **(G)** Representative images showing MCF-7 tumor size after treatment with the indicated conditions. Pooled data of tumor weight **(H)** expressed as fold change over 0 mT. TRPC1 **(I)** and Cyclin D1 **(J)** protein expression levels normalized to GAPDH and expressed as fold change over 0 mT. Measurements were minimally in triplicates, from minimally 6 independent eggs; *p < 0.05 and **p < 0.01 or as indicated. Error bars represent the standard error of the mean. **(K)** Representative TUNEL staining of isolated MCF-7 CAM tumors (n=4 per group) and corresponding violin-plots **(L)** of TUNEL fluorescence intensity. Two sections per tumor were analyzed using One-Way ANOVA and Sidak’s multiple comparisons test; **p < 0.01 and ^#^ *p* < 0.0001. **(M)** Representative micrographs of 5 chick livers per group showing TUNEL staining under the indicated conditions. Scale bar = 100 µm. TUNEL positive controls of MCF-7 tumor and liver sections were treated with DNAse prior to the staining with TUNEL. The nuclei were stained with DAPI (blue). “ns” indicates statistically nonsignificant differences.

The chicken chorioallantoic membrane (CAM) model was employed to initially explore the mechanisms conferring vulnerability of breast cancer to PEMF exposure (25). The absence of an immune system and the presence of estrogen and progesterone during early chick development (26, 27) make the CAM model an ideal host for the grafting of hormone-sensitive cells such as MCF-7 breast cancer cells (25). MCF-7-derived tumors were implanted into 7-day old eggs and allowed to engraft for 3 days before daily exposure to 3 mT PEMFs for 1 h per day for 4 consecutive days (**Figure 2A**). Chick embryos collaterally exposed to the fields did not show any intergroup weight differences by study termination (**Figure 2B**). On the other hand, tumor xenografts exposed to 3 mT PEMFs showed a substantial loss in mass (~50%) compared to unexposed (0 mT) tumors (**Figures 2C, D**). In parallel, TRPC1 channel protein expression was downregulated by ~30% in PEMF exposed tumors (**Figure 2E**), whereas Cyclin D1 levels were insignificantly affected (**Figure 2F**), suggesting that apoptosis, rather than a slowing of cell proliferation, is the predominant effect produced by magnetic field exposure. Combining the treatments (3 mT + DOX) reduced tumor weight by ~57% of control (0 mT + Saline), compared with ~36% observed with DOX alone (**Figures 2G, H**), corroborating previously observed synergism (**Figures 1B, D**). Combined 3 mT and DOX treatments also downregulated TRPC1 channel expression by ~40% (**Figure 2I**) and Cyclin D1 protein expression to ~50%, albeit not significantly (**Figure 2J**), of control values (0 mT + Saline). TUNEL staining of MCF-7 CAM tumors treated with PEMFs and DOX showed greater levels of DNA fragmentation than DOX treatment alone or control (**Figures 2K, L**). TUNEL staining performed on the livers of the chicken embryos following the distinct treatments revealed no significant changes in the number of cells exhibiting apoptotic DNA fragmentation (**Figure 2M**), reinforcing that the PEMF paradigm is specifically damaging to cancerous cells and not collateral tissues.

### PEMF and DOX Treatments Impair *In Vitro* and *Ex Vivo* Human Breast Cancer Cell Survival

**Figure 3:**
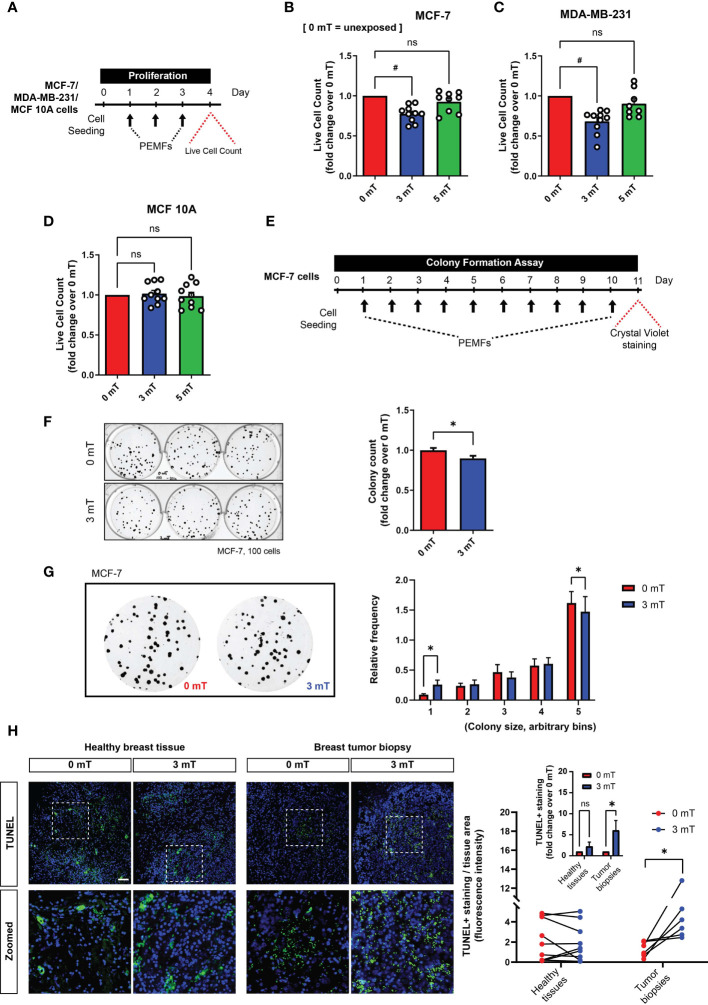
PEMF exposure inhibits cancer cell growth *in vitro* and *ex vivo* (human). **(A)** Schematic of PEMF exposure schedule for live cell quantification. Live cell counts using Trypan Blue exclusion assay for MCF-7 **(B)**, MDA-MB-231 **(C)** and MCF10A **(D)** cells. Cells were exposed to 0 mT, 3 mT, or 5 mT PEMF for 1 h each day for 3 consecutive days before cell quantification with ^#^ *p* < 0.0001. **(E)** Schematic of MCF-7 clonal colony formation regime. PEMF exposure was conducted at 3 mT for 1 h/day. Representative images of MCF-7 cell colonies formed with and without PEMF exposure (left) and associated quantification of surviving colonies shown as fold change over 0 mT (right). **(G)** Blown-up images of MCF-7 colonies formed with (3 mT) and without (0 mT) PEMF exposure (left) and corresponding colony size frequency distributions normalized to the total number of colonies (right). Cells were seeded at a density of 100 per well. Data generated from 3 to 6 independent biological replicates with *p < 0.05. The error bars represent the standard error of the mean. (**H**, left) TUNEL staining (green) of paired breast tumor biopsies or healthy breast tissues 24 h following 3 mT PEMF exposure or in the unexposed (0 mT) state. Nuclei were stained with DAPI (blue). Scale bar = 100 µm. (**H**, right) Corresponding line plot showing changes in TUNEL staining between paired samples exposed to 0 mT or 3 mT PEMFs per tissue area. Each line represents one independent patient sample divided into two parts for independent treatment and compared using one-tail paired *t*-test. Inset graph is the pooled average of the same TUNEL analysis after the normalization of 3 mT/0 mT of every individual sample, expressed as fold change over 0 mT. Analysis was performed using Two-Way ANOVA with Sidak’s multiple comparisons test, with *p < 0.05 and the error bars represent the standard error of the mean. ‘ns’ indicates statistically nonsignificant differences.

Corroborating previous findings (10), PEMF exposure (**Figure 3A**) reduced the number of viable MCF-7 (~22%; **Figure 3B**) and MDA-MB-231 (~32%; **Figure 3C**) breast cancer cells, whereas MCF10A normal breast cells were insensitive to the same exposure paradigm (**Figure 3D**). Notably, stronger PEMF exposures (5 mT) were ineffective at killing MCF-7 and MDA-MB-231 breast cancer cells (**Figures 3B**–**D**). A clonogenic assay was used to determine the *in vitro* long-term effects of magnetic field exposure on breast cancer cells (24), whereby MCF-7 cells were plated at clonal density and exposed to 3 mT PEMFs for 10 successive days (**Figure 3E**). Colony number (**Figure 3F**) and size (**Figure 3G**) were both reduced by PEMF exposure (blue) relative to unexposed (0 mT; red) cultures, consistent with a slowing of MCF-7 proliferation in response to PEMF exposure (**Figure 3B**). Moreover, the *ex vivo* examination of human breast tumor biopsies displayed increased TUNEL staining following a single PEMF exposure for 1 h, without any significant change in healthy breast tissues (**Figure 3H**), indicating the vulnerability of breast tumors to PEMF exposure.

**Figure 4:**
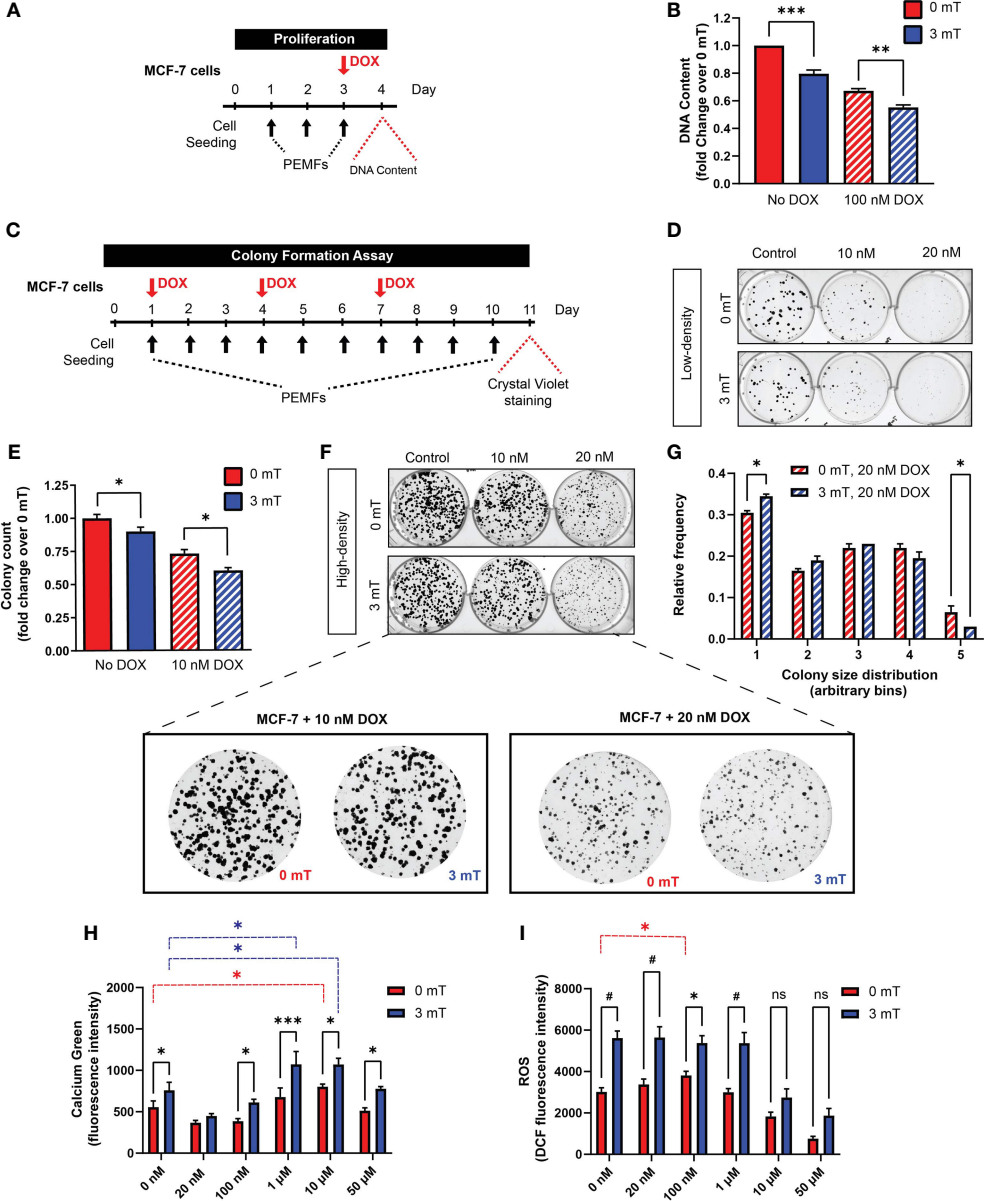
PEMF exposure enhances MCF-7 cell vulnerability to doxorubicin. **(A)** Schematic of PEMF and DOX treatment regime for DNA quantification. Cells were exposed to 3 mT for 1 h daily for 3 successive days. DOX (100 nM) was administered on the final day 1 h before the last PEMF exposure. Cellular DNA content was measured 24 h after the last PEMF exposure. **(B)** Quantification of pooled data for cellular DNA content represented as fold change 24 h post-DOX and PEMF treatments. **(C)** Colony formation paradigm for MCF-7 cells treated with DOX and PEMFs. **(D)** MCF-7 colony formation in the presence of 10 and 20 nM DOX, with and without PEMF exposure, as indicated. Cells were seeded at a density of 100 per well. **(E)** Colony survival in 10 nM DOX under low-density conditions. Colony survival in the presence of 20 nM DOX was too low to accurately quantify. **(F)** MCF-7 colony formation in the presence of 10 and 20 nM DOX, with and without PEMF exposure under high-density seeding condition of 1000 cells per well. (**F**, below) Zoom-in images of cells under the DOX treatment paradigm. **(G)** Colony size frequency distribution in the presence of 20 nM DOX and 0 mT or 3 mT exposure, indicated by the red and blue hatched bars, respectively; high density cultures. **(H)** Absolute calcium fluorescence intensity of MCF-7 cells treated with 16 h DOX at 20 nM, 100 nM, 1 μM, 10 μM and 50 μM. Cells were loaded with Calcium Green-1 and exposed to PEMFs for 30 min before rinsing, and fluorescence determination 25 min later. **(I)** Absolute DCH_2_FDA-ROS fluorescence from MCF-7 cells. Cells were similarly treated with DOX (20 nM, 100 nM, 1 µM, 10 µM and 50 µM) for 16 h before simultaneous incubation in DCH_2_FDA and exposure to PEMFs for 30 min. ROS measurement was performed every hour and data presented were from the 4^th^ hour. Calcium and ROS values represent the average of 7 technical replicates per condition. All data generated were from 3 to 5 independent experiments, with *p < 0.05, **p < 0.01, ***p < 0.001 and ^#^ *p* < 0.0001. The error bars represent the standard error of the mean. “ns” indicates statistically nonsignificant differences.

*In vitro* synergism between PEMF and DOX treatments was next investigated. Cell number was examined in response to 3 consecutive days of PEMF exposure (1 h/day) with DOX co-administered on the third day (**Figure 4A**) at the reported IC_50_ for MCF-7 cells (100 nM) (28). PEMF exposure alone reduced the cellular DNA content by ~20% (**Figure 4B**; solid blue), whereas DOX treatment alone reduced DNA content by ~30% (**Figure 4B**; hatched red) relative to unexposed control cultures (**Figure 4B**; 0 mT, solid red). The preconditioning of MCF-7 cells with two days of PEMF exposure accentuated DOX-cytotoxicity by ~45% (**Figure 4B**; hatched blue), recapitulating the *in vivo* scenarios (**Figure 1**; green and **Figure 2H**). The consequences of PEMF exposure during long-term DOX treatment were next ascertained with the clonogenic system by reducing the concentrations of DOX to 10 nM (10-fold) or 20 nM (5-fold). MCF-7 cultures were administered DOX on days 1, 4, and 7 in conjunction with daily PEMF exposure for a total of 10 days (**Figure 4C**). Colony number (**Figures 4D, E**) and size (**Figures 4F, G**) were noticeably decreased in response to DOX in low- and high-density cultures, respectively, at either dose. Individually, DOX (10 nM) (**Figure 4D**; top, middle) and PEMF (**Figure 4D**; bottom, left) treatments attenuated MCF-7 colony number by ~30% (**Figure 4E**; hatched red) and ~10% (**Figure 4E**; solid blue), respectively, whereas the combination DOX (10 nM) and PEMF exposure (**Figure 4D**; bottom, middle) reduced colony number by 40% (**Figure 4E**; hatched blue), relative to unexposed 0 mT cultures (**Figure 4D**; top, left and **Figure 4E**; solid red). The combination of DOX (20 nM) and PEMF exposure (**Figure 4G**; blue hatched) produced a general shift towards smaller colonies, increasing and decreasing the incidence of the smallest and largest colonies, respectively, relative to DOX-treatment alone (**Figure 4G**; red hatched). PEMF and DOX treatments hence synergize *in vitro* to slow breast cancer cell growth.

PEMF exposure stimulates ROS production in cancer (29, 30) and non-cancer (5, 31, 32) cells. Underlying this response is a magnetically-responsive, TRPC1-mediated calcium entry pathway that modulates mitochondrial respiration and cell proliferation (5, 32). By contrast, DOX increases cytoplasmic and mitochondrial ROS by disrupting mitochondrial redox cycling and function (33). Changes in cytoplasmic calcium were measured in MCF-7 cells for DOX concentrations ranging from 20 nM to 50 µM, with (**Figure 4H**; blue) and without (**Figure 4H**; red) PEMF exposure. PEMF exposure (blue) consistently increased cytoplasmic calcium over baseline (red) and was further augmented with increasing DOX concentration, reaching a maximum at 1 µM and then diminishing at 50 µM, likely reflecting increased cytotoxicity with higher DOX level. Analogously, PEMF exposure of MCF-7 cells (**Figure 4I**; blue) consistently increased ROS levels over baseline (0 mT, red) at all DOX concentrations. Moreover, basal calcium (**Figure 4H**; red) and ROS (**Figure 4I**; red) levels were augmented over baseline (0 nM DOX) at 10 µM and 100 nM DOX, respectively, providing mechanistic basis for potential synergism between the two treatments. PEMF and DOX treatments may hence synergize by raising cytoplasmic calcium and ROS levels in MCF-7 cells, consistent with an involvement of TRPC1, which has been shown to parallel MCF-7 malignancy status (5, 34).

### PEMF and DOX Treatments Target Breast Cancer Cells Exhibiting Elevated TRPC1 Levels

**Figure 5:**
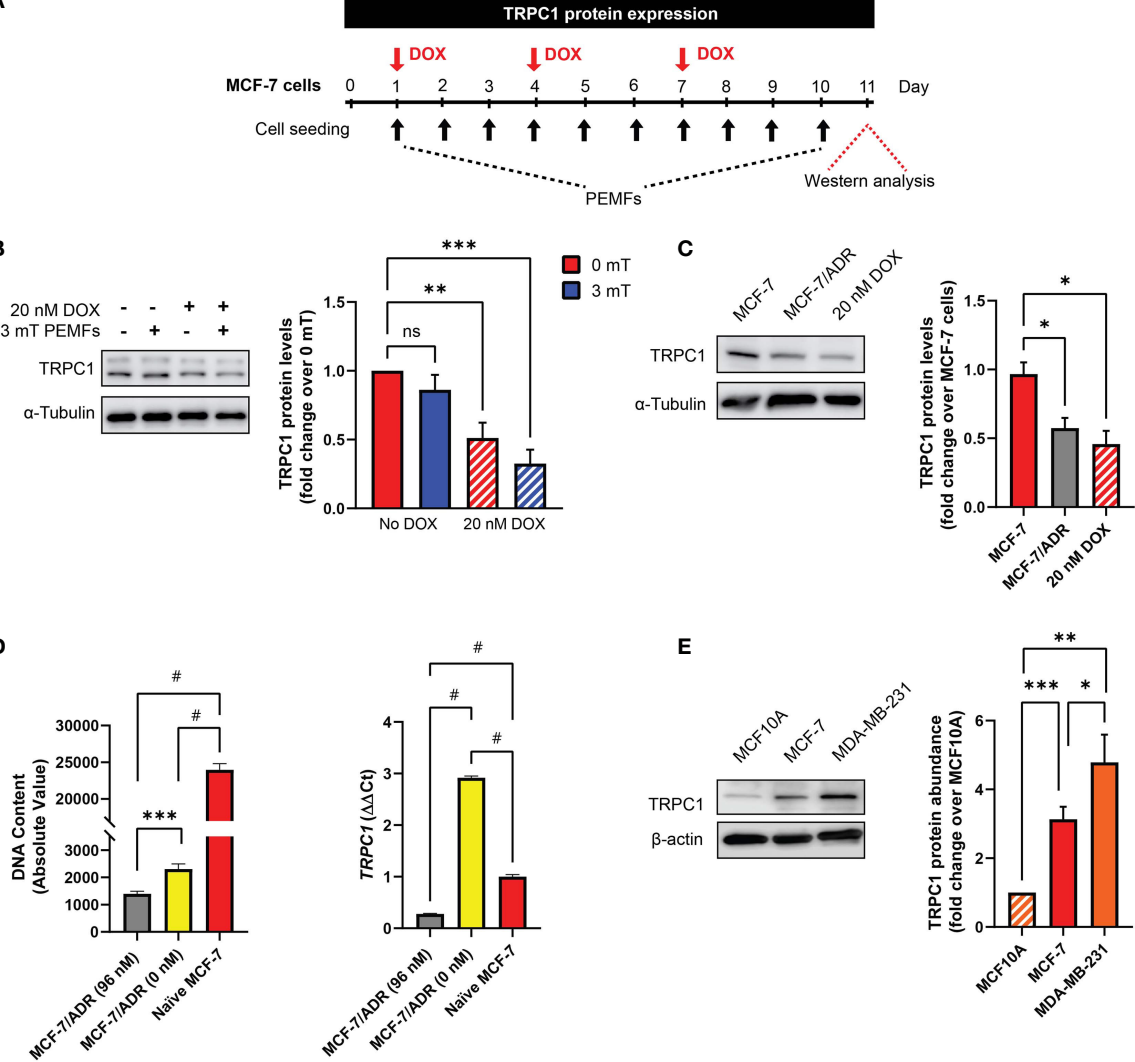
DOX-chemoresistance is associated with TRPC1 channel downregulation. **(A)** PEMF (black arrows, 3 mT) and DOX (red arrow, 20 nM) regime used for TRPC1 protein analysis. **(B)** Representative western blot showing changes in TRPC1 after 11 days under the indicated conditions and corresponding quantification of TRPC1 protein levels normalized to unexposed 0 mT. Cells treated with DOX are represented by the hatched bars in combination with either 0 mT (red) or 3 mT (blue) PEMF exposure. **(C)** Western blot analysis of TRPC1 protein levels in MCF-7/ADR (96 nM DOX; gray) and 10-day 20 nM DOX-treated MCF-7 cells (hatched red) relative to naïve MCF-7 cells (red). Naïve MCF-7 and MCF-7/ADR cells were grown in culture for 3 days before protein analysis. **(D)** Comparison of cell growth (72 h post-seeding; left) and *TRPC1* transcript levels (right) between MCF-7/ADR (96 nM), MCF-7/ADR (0 nM) and naïve MCF-7 cells. MCF-7/ADR (96 nM) cells were generated using incremental DOX levels up to a final concentration of 96 nM (gray). Serial passaging of MCF7/ADR (96 nM) cells in the absence of DOX gave rise to MCF-7/ADR (0 nM) cells (yellow). **(E)** Western analysis of TRPC1 protein levels in non-malignant (MCF10A) and malignant breast cancer (MCF-7 and MDA-MB-231) cells after 48 h of growth under standard conditions. The corresponding bar chart shows the pooled data in fold change of TRPC1 expression normalized to MCF10A. All results presented were from 3 to 5 independent experiments with *p < 0.05, **p < 0.01, ***p < 0.001 and *^#^p* < 0.0001. The error bars represent the standard error of the mean. “ns” indicates statistically nonsignificant differences.

TRPC1 channel expression was reduced in MCF-7 CAM tumors exposed to DOX and PEMF, alone or in combination (**Figure 2I**). To gain mechanistic insight into this response, the *in vitro* modulation of TRPC1 expression was examined following chronic PEMF and/or DOX exposures and recovery as well as in distinct breast cancer cell lines (**Figure 5A**). Chronic DOX treatment alone (**Figure 5B**; hatched red) reduced TRPC1 protein expression by ~50% of control levels (solid red) and was further reduced by an additional ~10% (hatched blue) when combined with daily PEMF exposure. By contrast, PEMF exposure alone was incapable of significantly reducing TRPC1 expression (solid blue). Growth under chronic and progressive DOX treatment (≤ 96 nM) moreover, produced a DOX-resistant MCF-7 cell line (MCF-7/ADR) (28) that exhibited both attenuated TRPC1 expression (~-40%) (**Figure 5C**; gray) and proliferation (~-90%) (**Figure 5D**; gray). MCF-7/ADR cells serially passaged (>5 times) in the absence of DOX, partially regained *TRPC1* expression as well as proliferative capacity (**Figure 5D**, yellow). These results accord with previous studies showing that chronic DOX exposure produces DOX-resistant MCF-7 cells (28). Therefore, chronic DOX exposure at the predetermined IC_50_ of native MCF-7 cells is capable of selecting against breast cancer cells with innately elevated TRPC1 expression to produce cellular progeny elaborating depressed TRPC1 expression, proliferative capacity and chemosensitivity. A relationship between DOX sensitivity and TRPC1 expression levels is hence revealed that reconciles previous findings showing that DOX targets proliferating MCF7 cells (35), a phenotype that is dependent on TRPC1 expression (5, 34). Finally, MDA-MB-231 (**Figure 5E**, orange) exhibited greater TRPC1 expression than MCF-7 cells (**Figure 5E**, red) aligning with their heightened vulnerability to PEMF exposure, manifested as 68% (+/- 4%, SEM) and 78% (+/- 3%, SEM) survival following PEMF exposure, respectively (**Figures 3C** *vs* **3B**).

### TRPC1 Overexpression Enhances MCF-7 Proliferation and EMT, but Attenuates Migratory Capacity

**Figure 6:**
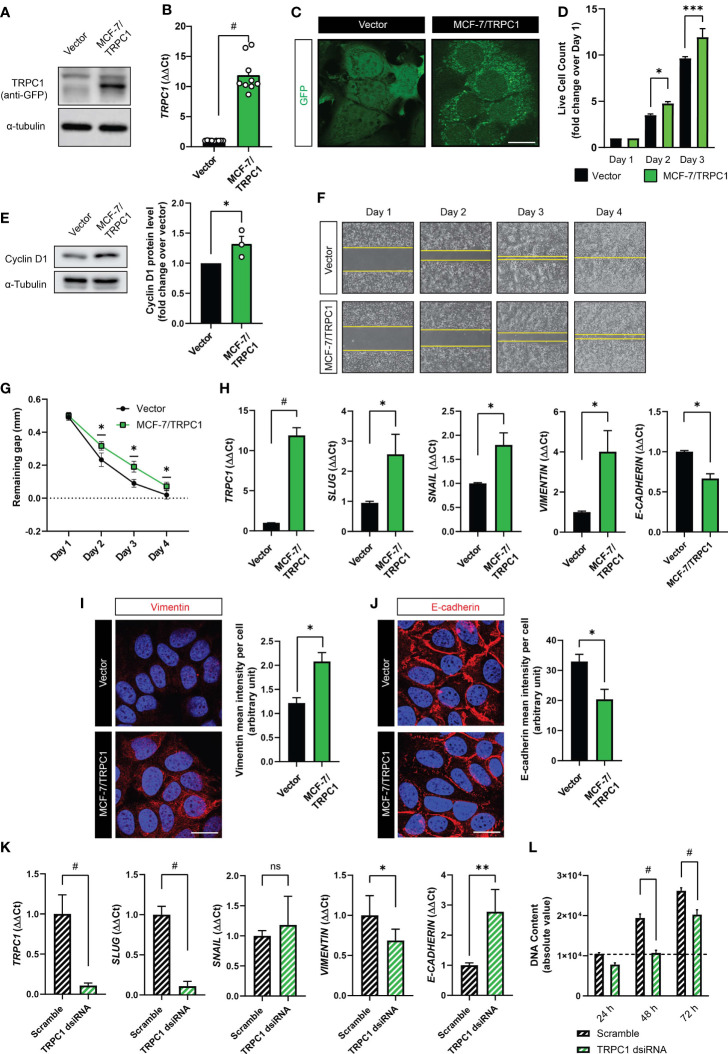
Characterization of TRPC1 overexpressing MCF-7 cell line. **(A)** Western analysis showing the overexpression of GFP-TRPC1 in MCF-7 cells, detected using anti-GFP antibody. **(B)** Quantification of ΔΔCt fold change of *TRPC1* transcript levels in MCF-7/TRPC1 cells (green) and vector-transfected cells (black). **(C)** Fluorescence images showing GFP and GFP-TRPC1 in vector and MCF-7/TRPC1 cells, respectively. Scale bar = 10 µm. **(D)** Live cell counts for MCF-7/TRPC1 (green) and vector-transfected (black) cells over 3 days. **(E)** Western analysis showing Cyclin D1 protein levels 24 h post-seeding. **(F)** Representative images of cell migration over 4 days. Stable cells were seeded at a density of 30,000 per well one day before the removal of the insert to create a 0.5 mm gap. **(G)** Gap remaining over 4 days of the migration assay. **(H)** Transcript levels of *TRPC1*, *SLUG*, *SNAIL*, *VIMENTIN*, and *E-CADHERIN* in vector (black) and MCF-7/TRPC1 (green) cells. Representative confocal images of vector and MCF-7/TRPC1 cells stained for Vimentin **(I)** and E-Cadherin **(J)** alongside corresponding histograms of mean intensity per cell. Absolute fluorescence intensity was normalized to the total number of nuclei per view. Scale bar = 10 μm. **(K)** Transcript levels for *TRPC1*, *SLUG*, *SNAIL*, *VIMENTIN*, and *E-CADHERIN* in scrambled- (black hatched) and TRPC1-silenced (green hatched) cells. **(L)** Proliferation over 3 days for *TRPC1*-silenced cells relative to scramble RNA-transfected cells. *TRPC1* silencing was achieved using 2 independent dsiRNAs and the bar charts show the respective quantification of the pooled data. All results were from 3 to 5 independent experiments with *p < 0.05, **p < 0.01, ***p < 0.001 and ^#^ *p* < 0.0001. The error bars represent the standard error of the mean. “ns” indicates statistically nonsignificant differences.

Potential mechanistic interactions between TRPC1 expression, proliferative and migratory capacities and epithelial-mesenchymal transition (EMT) were investigated (5, 31, 36–38). Towards this objective, a GFP-TRPC1 fusion protein overexpressing MCF-7 cell line (MCF-7/TRPC1) was generated and compared against a stable cell line expressing the GFP vector alone. The levels of GFP-TRPC1 fusion protein (**Figure 6A**), *TRPC1* transcripts (**Figure 6B**) and TRPC1 fluorescence staining (**Figure 6C**) were all much greater in the MCF-7/TRPC1 cells. The MCF-7/TRPC1 cells (**Figure 6D**; green) also exhibited enhanced proliferation (**Figure 6D**; black) consistent with a greater protein expression of Cyclin D1 (**Figure 6E**). On the other hand, the MCF-7/TRPC1 cells migrated more slowly (**Figure 6F**; bottom) than the vector control cells (**Figure 6F**; top), manifested as a delayed closure of an introduced gap (**Figure 6G**). TRPC1 overexpression also increased the gene expression of the EMT transcriptional activators involved in metastatic reprogramming (39), *SLUG*, and *SNAIL* (**Figure 6H**). Consistent with published findings (39), *SLUG* upregulation was associated with increased *VIMENTIN* (**Figure 6H**, transcript; **Figure 6I**, protein) and decreased E-cadherin (**Figure 6H**, transcript; **Figure 6J**, protein) levels. These results accord with previous reports of Slug and Snail transcriptional regulation of E-Cadherin (40) in breast cancer (41, 42). Conversely, *TRPC1* silencing resulted in the downregulation of *SLUG* and *VIMENTIN*, with corresponding increases in *E-CADHERIN*, while *SNAIL* levels remained unchanged (**Figure 6K**). The dsiRNA silencing of TRPC1 in naïve MCF-7 cells also reduced basal proliferation (**Figure 6L**), confirming TRPC1 as a proliferation modulator. The sum of these data provide evidence for TRPC1 involvement in breast cancer metastatic reprogramming.

### PEMF Exposure Slows the Migration and Decreases the Invasiveness of TRPC1-Overexpressing Breast Cancer Cells

**Figure 7:**
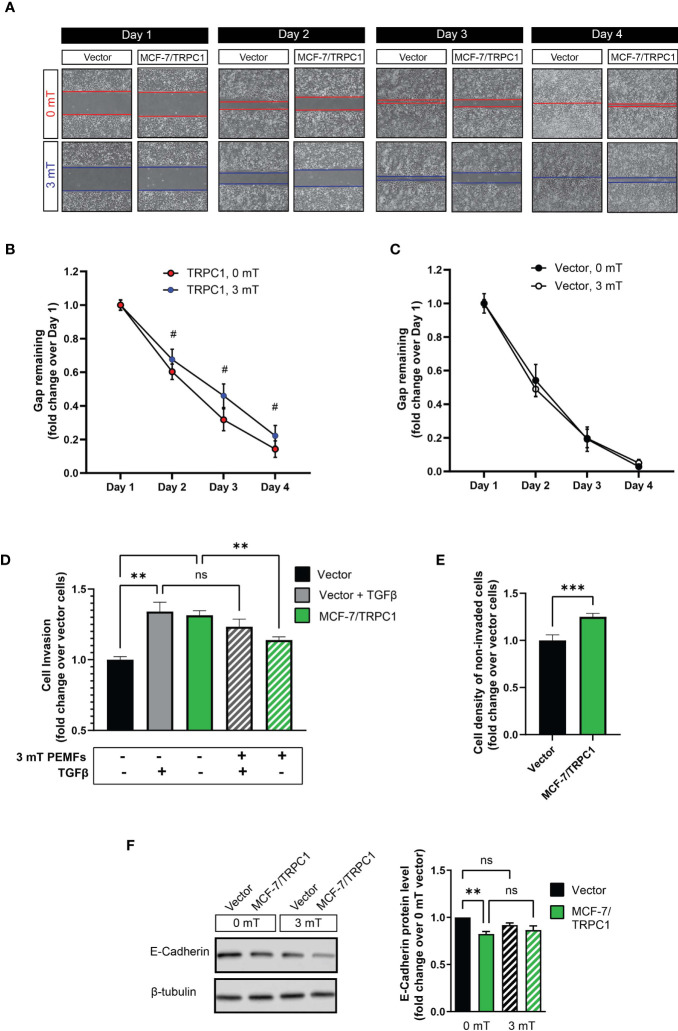
PEMF exposure attenuates migration and invasion of MCF-7/TRPC1 cells. **(A)** Photographic comparison of the migration of vector-transfected and MCF-7/TRPC1 cells exposed to 0 or 3 mT PEMFs. Cells were plated at a density of 30,000 per well and allowed to settle for 24 h before the removal of the insert. Cells were exposed to PEMFs for 1 h on the second, third and fourth days. Time course of gap closure for **(B)** MCF-7/TRPC1 (0 and 3 mT) and **(C)** vector (0 and 3 mT) cells normalized to day 1. **(D)** Quantification of stained (invading) cells expressed as fold change relative to vector cells. The stained cells correspond to those that successfully invaded the basal membrane and are present on the lower side of the membrane after 48 h. Untreated vector cells served as a reference of basal cell invasion (black). Gray bars represent vector cells that had been treated with TGFβ during seeding to promote invasion in combination with (hatched) and without (solid) 3 mT PEMF exposure at seeding and 24 h later. **(E)** Cell density on the upper side of the insert after 48 h post-seeding. The cells were stained and lysed using the same schedule as for the invasion assay. **(F)** Western analysis of E-cadherin protein expression in vector and MCF-7/TRPC1 cells with and without PEMF exposure for 3 consecutive days. E-cadherin protein levels shown as fold change relative to that of 0 mT of vector cells. All results were from 3 to 5 independent experiments with **p < 0.01, ***p < 0.001 and ^#^ *p* < 0.0001. The error bars are expressed as the standard error of the mean. “ns” indicates nonsignificant differences.

PEMF exposure (3 mT) further decelerated the migration of MCF-7/TRPC1 cells relative to their unexposed (0 mT) state (**Figures 7A, B**), whereas the migratory capacity of vector cells was unaltered by PEMF exposure (**Figures 7A, C**). Invasiveness was ascertained by examining the ability of cells to break down, penetrate, and transverse a basement membrane-coated insert (**Figure 7D**). MCF-7/TRPC1 cells exhibited a level of invasiveness (**Figure 7D**; green), comparable in magnitude to TGFβ-stimulated control cells (gray), yet exceeding that of unstimulated vector cells (black). PEMF exposure attenuated the invasive capacity of MCF-7/TRPC1 cells (hatched green), but was incapable of doing so for TGFβ-stimulated vector cells (hatched gray). As the enhanced basal invasiveness of MCF-7/TRPC1 was accompanied by an increase in the number of non-invading cells on the upper side of the culture insert (**Figure 7E**), a causal relationship may exist between TRPC1-mediated enhancement of proliferation (**Figure 6D**) and invasiveness. On the other hand, the finding that PEMF exposure reduced invasiveness by attenuating both cell proliferation and slowing migratory capacity is clinically relevant. Agreeing with demonstrated transcriptional inhibition of E-cadherin in response to elevations in *SNAIL* and *SLUG* (**Figures 6H, J**), E-cadherin protein levels were found to be reduced in MCF-7/TRPC1 cells (**Figure 7F**). However, E-cadherin levels were unchanged by PEMF exposure (**Figure 7F**), possibly reflecting an offsetting combination of metastatic reversal and a PEMF-induced slowing of cell migration (**Figures 7A, B**), reinstating E-cadherin levels (43).

### TRPC1 Overexpression Increases Breast Cancer Cell Vulnerability to DOX and PEMFs

**Figure 8:**
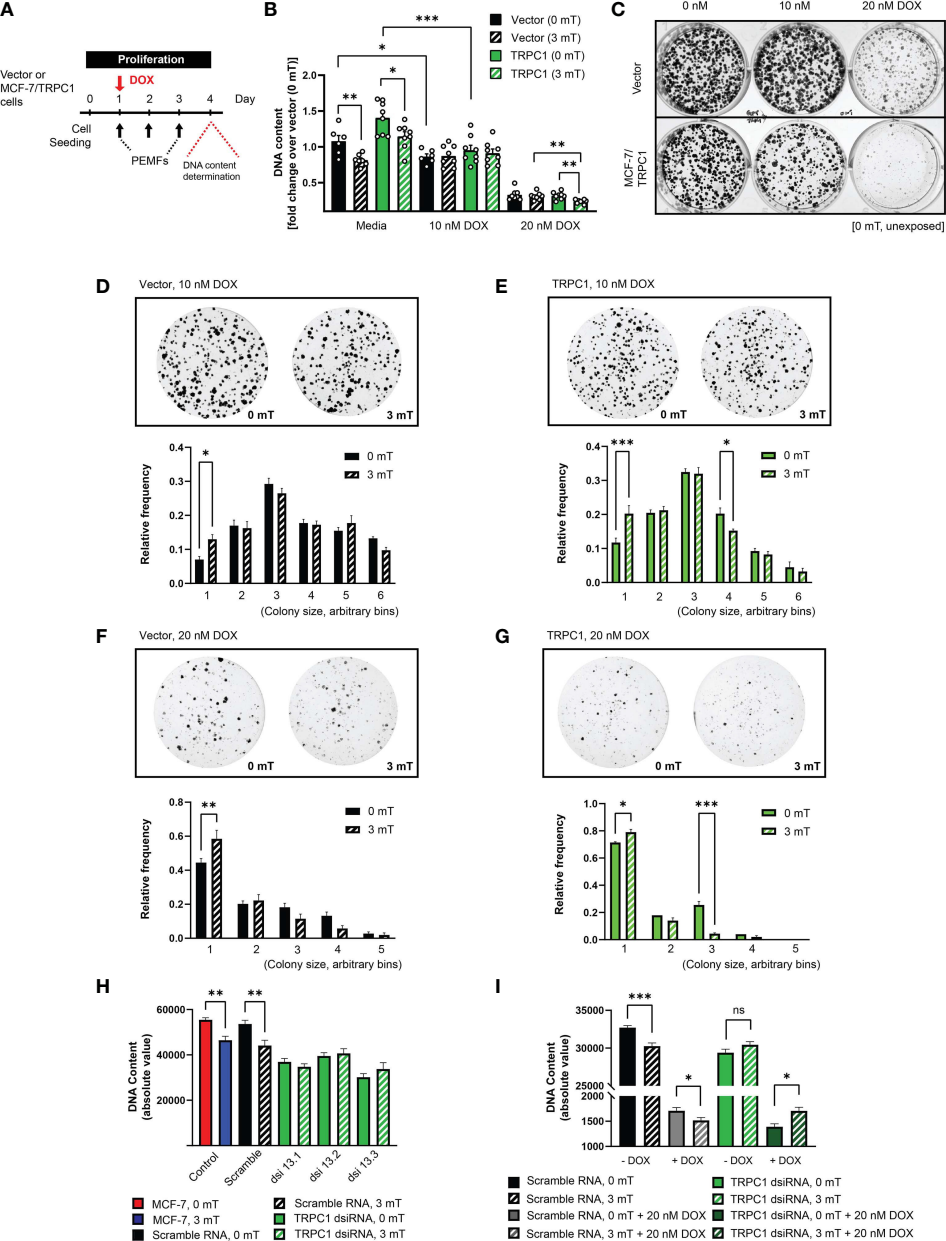
TRPC1 overexpression sensitizes MCF-7 cells to doxorubicin and PEMF exposure. **(A)** PEMF and DOX treatment regime for MCF-7/TRPC1 cellular DNA quantification. **(B)** Quantification of DNA fold change relative to 0 mT vector cells. Statistical analysis was done using the two-sample *t*-test. **(C)** Colony formation after 10 days in the presence of 0 nM, 10 nM or 20 nM DOX, without PEMF exposure. Images of MCF-7 colonies and corresponding colony size frequency distributions normalized to the total number of colonies in the presence of 10 nM DOX for **(D)** vector and **(E)** MCF-7/TRPC1 cells or 20 nM DOX for **(F)** vector and **(G)** MCF-7/TRPC1 cells, concomitant with daily PEMF exposure. *p < 0.05, **p < 0.01 and ***p < 0.001 indicate the statistical difference between the respective 0 mT and 3 mT condition within the same bin. **(H)** PEMF-modulated growth of *TRPC1*-silenced MCF-7 cells 48 h post dsiRNA transfection. Cells were transfected with three independent dsiRNA (green), including a scramble RNA (black). Control (untransfected) MCF-7 cells were exposed to 0 mT (red) or 3 mT (blue) PEMFs. **(I)** Combined effects of PEMF and DOX (20 nM) treatments on the proliferation of *TRPC1*-silenced cells. Cells were exposed to PEMFs 24 h and 48 h post dsiRNA transfection before DNA content analysis (hatched bars). The data for *TRPC1* dsiRNA (green and dark green) was pooled data from two independent *TRPC1* dsiRNAs. The statistical analysis was generated using Multiple unpaired *t*-test for the comparison of two sample means within the same colony size. All experiments were from 3 to 5 independent experiments with *p < 0.05, **p < 0.01 and ***p < 0.001. “ns” indicates statistically non-significant differences. The error bars represent the standard error of the mean.

Given reduced TRPC1 expression and induced DOX-resistance by chronic DOX exposure (**Figures 5B, C**), we examined whether forced TRPC1 expression would instead enhance sensitivity to DOX and/or PEMF exposure. MCF-7/TRPC1 cells were exposed to DOX (10 or 20 nM) for 4 days, with or without PEMF exposure for 3 days (**Figure 8A**). PEMF exposure *per se* mitigated cell growth of both MCF-7/TRPC1 (**Figure 8B**; green, solid versus hatched) and vector (black, solid versus hatched) cells. Notably, 10 nM DOX depressed the growth of both MCF-7/TRPC1 (~30%) and vector cells (~20%) without obvious response to PEMF exposure (**Figure 8B**). On the other hand, 20 nM DOX produced strong proliferation depressions of both MCF-7/TRPC1 (~80%) and vector (~70%) cells that was significantly augmented by PEMF exposure in MCF-7/TRPC1 (~85%).

Despite TRPC1 overexpression *per se* exerting only modest effects on MCF-7 colony formation (**Figure 8C**, left column), overexpression noticeably heightened MCF-7 vulnerability to both 10 nM (**Figure 8C**, middle column) and 20 nM DOX (**Figure 8C**, right column), resulting in fewer and smaller colonies than unexposed vector cultures (0 mT). Examining the effects of PEMF exposure on colony size for the vector control (**Figures 8D, F**) and MCF-7/TRPC1 (**Figures 8E, G**) cells in the presence of 10 nM (**Figures 8D, E**) and 20 nM (**Figures 8F, G**) DOX revealed that TRPC1 overexpression promoted synergism between the treatments that that was greatest in the presence of 20 nM DOX and was manifested as more pronounced shifts towards smaller colonies. By contrast, MCF-7 cells in which *TRPC1* had been genetically silenced (**Figure 8H**; green, dsiRNAs) exhibited reduced proliferation and were insensitive to PEMF exposure (hatched green), whereas untransfected (blue) or scramble RNA transfected cells (hatched black) showed the typical proliferation depression in response to PEMF exposure. Moreover, PEMF and DOX (20 nM) anti-cancer synergism was absent in the *TRPC1*-silenced cells (solid and hatched dark green), but persisted in the scramble RNA-transfected cells (**Figure 8I**; solid and hatched gray). These results confirm that TRPC1 channel expression level bestows synergistic capabilities to DOX- and PEMF-treatments.

## Discussion

Initial *in vitro* findings alluded to the capacity of pulsing magnetic fields to stymie MCF-7 breast cancer growth, without damaging non-malignant MCF-10 cells (10). Here, these earlier findings were substantiated in the *in vivo* CAM model as a host for MCF-7-derived tumors. Consistent with our *in vitro* findings (**Figure 3**) (10), analogous PEMF exposure significantly reduced tumor weight and size (**Figure 2D**) associated with a sparing of collateral tissues (**Figure 2M**). *Ex vivo* examination of human biopsies also demonstrated a selective vulnerability of breast tumors to PEMF exposure, whereas healthy breast tissues were sparred by the same exposure protocol (**Figure 3H**). Furthermore, the same magnetic field paradigm was also shown effective at significantly regressing the growth of human-derived tumors engrafted into a PDX-mouse model (**Figure 1B**), without implicating healthy tissues (**Figure 1D**). Robust specificity of our magnetic field paradigm for cancer has thus been initially demonstrated.

Synergism between PEMF and DOX treatments at slowing breast cancer tumor growth was demonstrated in both the CAM chicken and PDX mouse models. *In vitro*, synergism between PEMF exposure and DOX administration was demonstrated in acute and chronic paradigms, both demonstrating enhanced depressions in proliferation (**Figure 4B**) and colony-growth (**Figures 4D–G**), compared to either treatment alone. Individually, PEMF (5) and DOX (3) treatments induced increments in cytosolic calcium and ROS that synergized when combined. The cumulative oxidative stress produced by combined PEMF and DOX treatments may summate to create a sufficiently critical oxidative environment to slow breast cancer cell growth. PEMF exposure also synergized with pemetrexed and cisplatin in the acute paradigm, although with lower combined efficacy than with DOX (**Supplementary Figure 2A, B**).

In NSG mice hosting human breast tumors, the greatest reductions in patient-derived tumor size coincided with the largest increase in apoptosis and were observed by preceding the 3 weeks of DOX chemotherapy with 2 weeks of PEMF exposure. We further examined the effects of PEMF exposure on MDA-MB-231 and MCF-7 breast cancer cells engrafted into NSG mice employing a clinical prototype of a magnetic field device intended for eventual use in human breast cancer (**Supplementary Figure 1**). MDA-MB-231 tumors from NSG mice exposed once (3 mT x 1) or twice (3 mT x 2) to PEMFs exhibited increases in apoptosis of +11% and +34% over baseline (0 mT), respectively (**Supplementary Figures 3A, B**). Livers harvested from these same mice did not show any significant increase in apoptosis (**Supplementary Figure 3C**). MCF-7 xenografts were also subjected to the same PEMF/DOX paradigm previously employed in **Figure 1** (**Supplementary Figure 4A**). Again, PEMF and DOX treatments synergized to enhance cancer cytotoxicity, achieving +26% and +33% apoptosis (early plus late) for tumors subjected to paradigms 1 (PEMF then DOX) and 2 (PEMF and DOX), respectively (**Supplementary Figure 4B**), and were greater than those achieved with DOX (+15%) or PEMF (+8%) treatments alone, whereas baseline (0 mT) exhibited negligible apoptosis (+0.3%). As in the PDX mouse trial (**Figure 1**), synergism between DOX and PEMF treatments was evident in undermining *in vivo* cancer growth, apparently without implicating healthy tissues. In humans, magnetic therapy will offer the advantage of being targetable to a body region inflicted with cancer for localized synergism with systemic DOX administration (**Supplementary Figure 1**), potentially allowing for the lowering of chemotherapeutic dose and reducing the severity of collateral cytotoxic DOX-TRPC channel interactions, such as doxorubicin-induced cardiotoxicity (44).

### Magnetic Mitohormesis in Cancer

Mitohormesis refers to a developmental process whereby low levels of ROS prime mitochondrial survival adaptations by augmenting the cell’s antioxidant defenses, whereas exaggerated levels of oxidative stress instead overwhelm the cell’s existing antioxidant defenses to stymie survival (7). TRPC1 was shown to be necessary and sufficient to confer mitochondrial responses to magnetic fields, creating the possibility of invoking a novel process of Magnetic Mitohormesis in TRPC1 expressing cells (5, 11). With reference to this study, PEMF exposure was shown to undermine MCF-7 and MDA-MB-231 cell growth (**Figures 3B, C**) in correlation with TRPC1 expression (**Figure 5E**), presumably due to the over-stimulation of this recently elucidated calcium-mitochondrial axis (**Figure 4H**) (5, 11). Previously it was shown that magnetosensitivity and downstream mitochondrial responses correlate with TRPC1 developmental expression and function (5, 11, 32, 45), whereas other common TRP channels did not show such a strong correlation (5, 32, 45). Moreover, the genetic silencing of TRPM7 (5), the most highly and ubiquitously expressed of all TRP channels (18), was unable to preclude magnetic sensitivity, whereas the genetic silencing of TRPC1 alone was capable of negating magnetic responsiveness. Finally, selective vesicular delivery of TRPC1 to cells genetically-engineered to be deficient in TRPC1 expression was sufficient to reinstate Magnetic Mitohormesis (5, 11). The magnetic sensitivity conferred by TRPC1 and its relevance to mitohormetic survival mechanisms (5, 11, 32) makes it a valuable target for clinical exploitation in cancer treatment (34).

### TRPC Channels in Breast Cancer

Diverse TRP channel classes have been broadly implicated in the development of various cancers in particular, TRPC1 in the realm of breast cancer (19, 36, 37, 46–48). Here, we show that TRPC1 overexpression increases MCF-7 proliferation and sensitivity to DOX yet, reduces migratory capacity (**Figures 6**–**8**). In a similar manner, the overexpression of miR-146b, an inflammatory modulator (49), enhanced the proliferation and chemosensitivity of epithelial ovarian carcinoma cells to cisplatin and paclitaxel while attenuating migratory capacity (50). Therefore, under pro-inflammatory conditions, such as those induced with the overexpression of TRPC1 or miR-146b, the proliferative capacities and chemosensitivities of certain cancers increase, whereas migratory capacities diminish. Provocatively, these dichotomous proliferative and migratory responses to inflammatory conditions may represent a point of vulnerability to be exploited by PEMF-based therapies in order to mitigate the invasiveness of breast cancer cells. PEMF exposure attenuates the proliferative and migratory capacities of breast cancer cells in positive correlation to TRPC1 channel expression (**Figures 5** and **7**), aligning with evidence that catalytic activation of TRPC6, similarly implicated with proliferation and inflammatory responses in breast cancer, attenuates MDA-MB-231 breast cancer cell viability and migratory capacity (51). Both studies further demonstrated reductions in breast cancer cell invasiveness in response to activation of either TRPC1 (PEMF exposure) (**Figure 7D**) or TRPC6 (Furin inhibition) (51). Moreover, TRPC1 channel expression is greater in the highly metastatic MDA-MB-231 cell line compared to the less invasive MCF-7 cell line (**Figure 5E**) (52). These findings demonstrate the value of inducing TRPC-mediated inflammatory responses for the targeted purpose of attenuating breast cancer invasiveness and allude to a therapeutic niche for PEMF-based therapies in cancer treatment. Our future studies will entail the examination of the contribution of other TRPC channel classes in breast cancer progression as well as the evaluation of their utility for potential exploitation within the PEMF-DOX synergistic anti-cancer axis.

**Figure 9:**
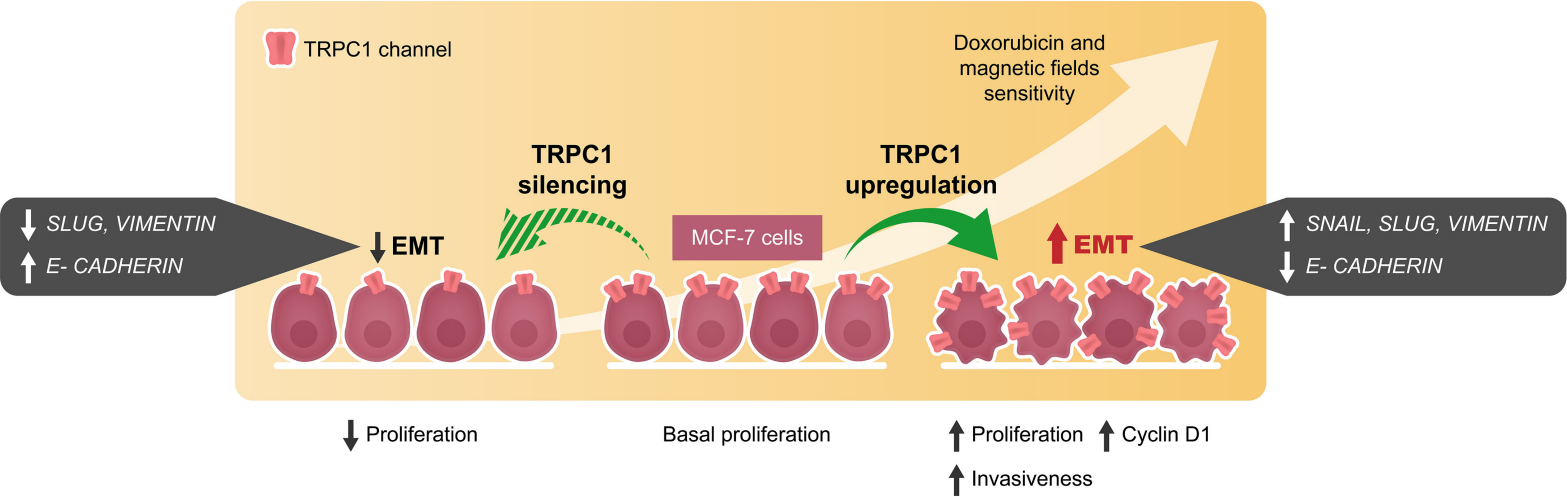
Schematic representation of cellular events modulated by the overexpression and silencing of TRPC1 in MCF-7 breast cancer cells. EMT and invasiveness were shown to correlate with TRPC1 expression in breast cancer cells providing an opportunity for the development of synergistic companion magnetic and doxorubicin therapies that selectively target TRPC1 for the treatment of cancer classes characterized by elevated expression of TRPC1.

EMT is the multifaceted process whereby transformed cells acquire metastatic capabilities and resistance to apoptosis (40, 53). The higher TRPC1 expression found in small histological grade 1 breast tumors, relative to larger grade 3 tumors (19), is related to their greater propensity to undertake EMT (36, 54). In accordance, we demonstrate that overexpression of TRPC1 in MCF-7 cells upregulated the expressions of *SLUG*, *SNAIL*, and *VIMENTIN* and downregulated the expression of E-cadherin in (**Figures 6H**–**J**), consistent with metastatic induction (39). Conversely, TRPC1-silencing reduced the expressions of *SLUG* and *VIMENTIN* and upregulated *E-CADHERIN* (**Figure 6K**). Elevations of TRPC1 expression are common in breast cancer (19, 36, 38) and may serve to predispose pre-neoplastic cells towards EMT by conferring a more proliferative and invasive phenotype (**Figure 9**). The capacity of PEMF-based therapies to mitigate proliferation and invasiveness of breast cancer cells by selectively targeting cells with high TRPC1 channel expression thus merits future investigation in human clinical trials.

Indications of cytotoxic synergism between DOX and diverse TRPC channels exist (44). PEMF exposure stimulates TRPC1-mediated engagement of a calcineurin-NFAT signaling axis involved in mitochondrial homeostasis (5). In certain cancers, TRPC1 hyperactivity (55) may overwhelm NFAT-mediated mitochondrial homeostatic mechanisms (56), ultimately selecting against cancer cells with inherently high TRPC channel expression. Negative selection by DOX against cancer cells with elevated TRPC1 expression may contribute to the commonly described chemotherapy paradox (57), hallmarked surviving cancer cells with heightened chemoresistance. Accordingly, we showed that chronic exposure of MCF-7 cells to DOX, attenuated TRPC1 expression (**Figures 5B, C**) and produced DOX-resistant cells exhibiting slowed proliferation (**Figure 5D**) and lost responsiveness to PEMF exposure (**Supplementary Figure 5**). Analogously, genetic silencing of TRPC1 in MCF-7 cells mitigated proliferation (**Figures 8H, I**) and precluded responses to both PEMF (**Figure 8H**) and DOX (**Figure 8I**) treatments, suggesting a causal interaction of DOX and PEMFs with TRPC1. Serial passaging of MCF-7/ADR cells in the absence of DOX ultimately restored TRPC1 transcript levels (**Figure 5D**, right), proliferative capacity (**Figure 5D**, left) and sensitivity to PEMF exposure (**Supplementary Figure 5**; left). The reintroduction of either MCF-7/ADR (96 nM DOX) or MCF-7/ADR (0 nM DOX) cells into high doses of DOX (100 nM), however, precluded response to PEMF exposure (**Supplementary Figure 5**; right). Although the results provided in this study are internally consistent and provide initial proof of efficacy, the implications of the interplay between DOX-PEMF-TRPC1 needs to be better understood in actual clinical settings. The clinical elaboration of PEMF-based therapies may ultimately permit the lowering of DOX chemotherapeutic load to help avert collateral cytotoxicity (3) and paradoxical effects (57) associated with high clinical doses of DOX.

## Conclusions

TRPC1 is a mitohormetic determinant governing cellular inflammatory status and survival (5, 6, 11). Elevated TRPC1 expression defines numerous types of cancers (15, 37). We demonstrate that the vulnerability of breast cancer to PEMF and DOX therapies is positively correlated with TRPC1 expression as are invasiveness and EMT (**Figure 9**), conferring upon them a heightened level of specificity for TRPC1-characterized cancers. Importantly, as many cancers exist near the threshold of metabolic cytotoxicity, where even moderate enhancements in cellular metabolism are sufficient to cause homeostatic disequilibrium (8), the oxidative synergism demonstrated by the TRPC1-PEMF-DOX axis may be exploited to selectively induce cancer-specific metabolic catastrophe (**Figure 2**). The presented combinational therapeutic strategy may hence prove more selective and safer than conventional therapies, particularly for cancers characterized by elevated TRPC1 channel expression. Finally, given the demonstrated specificity of PEMF treatment for TRPC1 expression reported here and elsewhere (5, 11, 32, 45), complementation of conventional DOX-based chemotherapy by localizable PEMF exposure may serve as a method to avert collateral toxicity (3) as well as paradoxical effects (57), by allowing the lowering of dose systemically-delivered chemotherapeutic drug while maintaining a unique level of specificity for TRPC1-associated cancers. The potential value of these unique combination of features merit future investigation and clinical validation.

## Data Availability Statement

The original contributions presented in the study are included in the article/**Supplementary Material**. Further inquiries can be directed to the corresponding author.

## Ethics Statement

Patient samples were collected based on National Healthcare Group Domain Specific Review Board approval (2014/01088). The patients/participants provided their written informed consent to participate in this study. The animal study was reviewed and approved by Nanyang Technological University (NTU) Institutional Animal Care and Use Committee approval (ARF-SBS/NIE-A0141AZ, A0250AZ, A0324, and A0321).

## Author Contributions

YT, AF-O, and NT conceived and designed this study. YT, KC, CF, and SR performed the cellular proliferation, ROS, RNA and protein analyses. YT, CF, JLY, and JNY generated and characterized the MCF-7 stable cell line overexpressing TRPC1. YT, CF, KC, and SR performed colony-forming assays, migration and invasion assays. RH and AK established the CAM model. KC, SR, YT, and CF performed the MCF-7 cells on CAM model. CC provided clinical human breast tumors. YY and WT performed the histology on clinical samples and pre-clinical PDX model using human breast tumors and cell lines. YT, KC, CF, SR, NT, and AF-O compiled and analyzed the data. JF designed the breast coil device used in some of the studies. JL managed the fabrication of the breast coil with the contract manufacturer, FLEX Ltd (Singapore). YT and AF-O wrote the manuscript. All authors approved the final manuscript.

## Funding

This work is supported by Lee Kong Chian MedTech Initiative, Singapore (N-176-000-045-001), SMART Ignition Grant (R-176-000-206-592) and the Institute for Health Innovation & Technology, iHealthtech, at the National University of Singapore. The publication cost of this article is funded by Department of Surgery, Yong Loo Lin School of Medicine, National University of Singapore.

## Conflict of Interest

AF-O and JF are inventors on patent WO 2019/17863 A1, System, and Method for Applying Pulsed Electromagnetic Fields as well as are contributors to QuantumTx Pte. Ltd., which elaborates electromagnetic field devices for human use. JF was employed by Fields at Work GmbH. JL is currently an employee of QuantumTx Pte. Ltd.

The remaining authors declare that the research was conducted in the absence of any commercial or financial relationships that could be construed as a potential conflict of interest.

## Publisher’s Note

All claims expressed in this article are solely those of the authors and do not necessarily represent those of their affiliated organizations, or those of the publisher, the editors and the reviewers. Any product that may be evaluated in this article, or claim that may be made by its manufacturer, is not guaranteed or endorsed by the publisher.
