# Corrigendum: Modulated TRPC1 Expression Predicts Sensitivity of Breast Cancer to Doxorubicin and Magnetic Field Therapy: Segue Towards a Precision Medicine Approach

**DOI:** 10.3389/fonc.2022.892408

**Published:** 2022-04-20

**Authors:** Yee Kit Tai, Karen Ka Wing Chan, Charlene Hui Hua Fong, Sharanya Ramanan, Jasmine Lye Yee Yap, Jocelyn Naixin Yin, Yun Sheng Yip, Wei Ren Tan, Angele Pei Fern Koh, Nguan Soon Tan, Ching Wan Chan, Ruby Yun Ju Huang, Jing Ze Li, Jürg Fröhlich, Alfredo Franco-Obregón

**Affiliations:** ^1^ Department of Surgery, Yong Loo Lin School of Medicine, National University of Singapore, Singapore, Singapore; ^2^ Biolonic Currents Electromagnetic Pulsing Systems Laboratory (BICEPS), National University of Singapore, Singapore, Singapore; ^3^ Lee Kong Chian School of Medicine, Nanyang Technological University Singapore, Singapore, Singapore; ^4^ Cancer Science Institute of Singapore, National University of Singapore, Singapore, Singapore; ^5^ School of Biological Sciences, Nanyang Technological University Singapore, Singapore, Singapore; ^6^ Division of General Surgery (Breast Surgery), Department of Surgery, National University Hospital, Singapore, Singapore; ^7^ Division of Surgical Oncology, National University Cancer Institute, Singapore, Singapore; ^8^ Department of Obstetrics & Gynaecology, Yong Loo Lin School of Medicine, National University of Singapore, Singapore, Singapore; ^9^ Fields at Work GmbH, Zürich, Switzerland; ^10^ Institute of Electromagnetic Fields, ETH Zürich (Swiss Federal Institute of Technology in Zürich), Zürich, Switzerland; ^11^ Institute for Health Innovation & Technology (iHealthtech), National University of Singapore, Singapore, Singapore; ^12^ Competence Center for Applied Biotechnology and Molecular Medicine, University of Zürich, Zürich, Switzerland; ^13^ Department of Physiology, Yong Loo Lin School of Medicine, National University of Singapore, Singapore, Singapore; ^14^ Healthy Longevity Translational Research Programme, Yong Loo Lin School of Medicine, National University of Singapore, Singapore, Singapore

**Keywords:** breast cancer, PEMFs, EMT, patient-derived xenograft, chorioallantoic membrane, doxorubicin, TRPC1, chemotherapy

In the original article, there was a nonconsequential error in **Figure 1** as published. A file concatenation error occurred within the FACS analysis software that implicated the scatter dot-plots shown in **Figure 1C**. The corrected **Figure 1** appears below.

**Figure 1:**
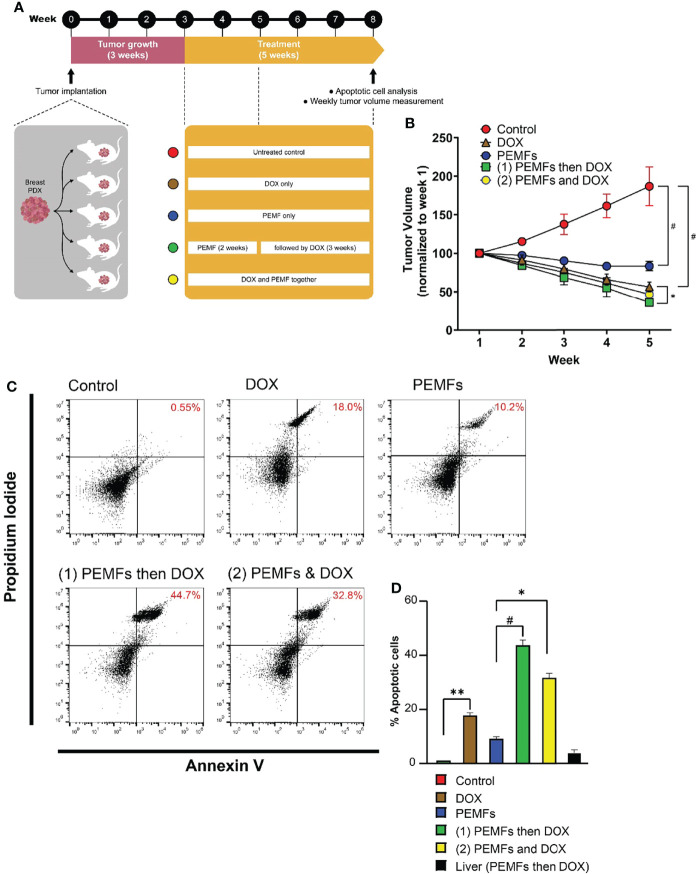
PEMFs synergize with DOX to inhibit tumor growth in vivo. **(A)** Schematic of PEMF and DOX exposure regimes used on mice hosting patient-derived tumor xenografts. Implanted tumors were allowed to grow for 3 weeks before the initiation of DOX (20 mg/kg) and/or PEMF treatments. Tumor volumes were measured each week while apoptotic cell determination was performed at the end of the study. Each data point represents the mean values from 5 experimental runs derived from the tumors obtained from 5 patients, each of which was equally divided amongst the 5 treatment groups. **(B)** Changes in tumor volume (mm^3^) for 5 weeks. **(C)** Representative scatter dot-plots showing cell populations from dissociated tumors based on Annexin V and propidium iodide staining. **(D)** Quantification of apoptotic cell percentages obtained using flow cytometry. *p < 0.05, **p < 0.01, and ^#^ *p* < 0.0001. Error bars represent the standard error of the mean.

The associated corrected values that appear in the text of the **Results** under the subheading “*PEMF and Doxorubicin Treatments Act Synergistically to Impair Breast Cancer Tumor Growth In Vivo*”, paragraph one, should read “Moreover, the incidence of apoptotic cells increased by +0.55%, +10.2%, and +18% in tumors isolated from control, PEMF-exposed and DOX-treated mice, respectively (**Figures 1C, D**)”.

The authors apologize for these errors and state that this does not change the scientific conclusions of the article in any way. The original article has been updated.

## Publisher’s Note

All claims expressed in this article are solely those of the authors and do not necessarily represent those of their affiliated organizations, or those of the publisher, the editors and the reviewers. Any product that may be evaluated in this article, or claim that may be made by its manufacturer, is not guaranteed or endorsed by the publisher.

