# Supplementary material for: Modulated TRPC1 Expression Predicts Sensitivity of Breast Cancer to Doxorubicin and Magnetic Field Therapy: Segue Towards a Precision Medicine Approach
Source: Front Oncol. 2022 Jan 24;11:783803. doi: 10.3389/fonc.2021.783803 (PMC8818958; doi:10.3389/fonc.2021.783803)
Supplement: Supplementary file 1 [file DataSheet_1.docx]

**Supplementary Figures**

**
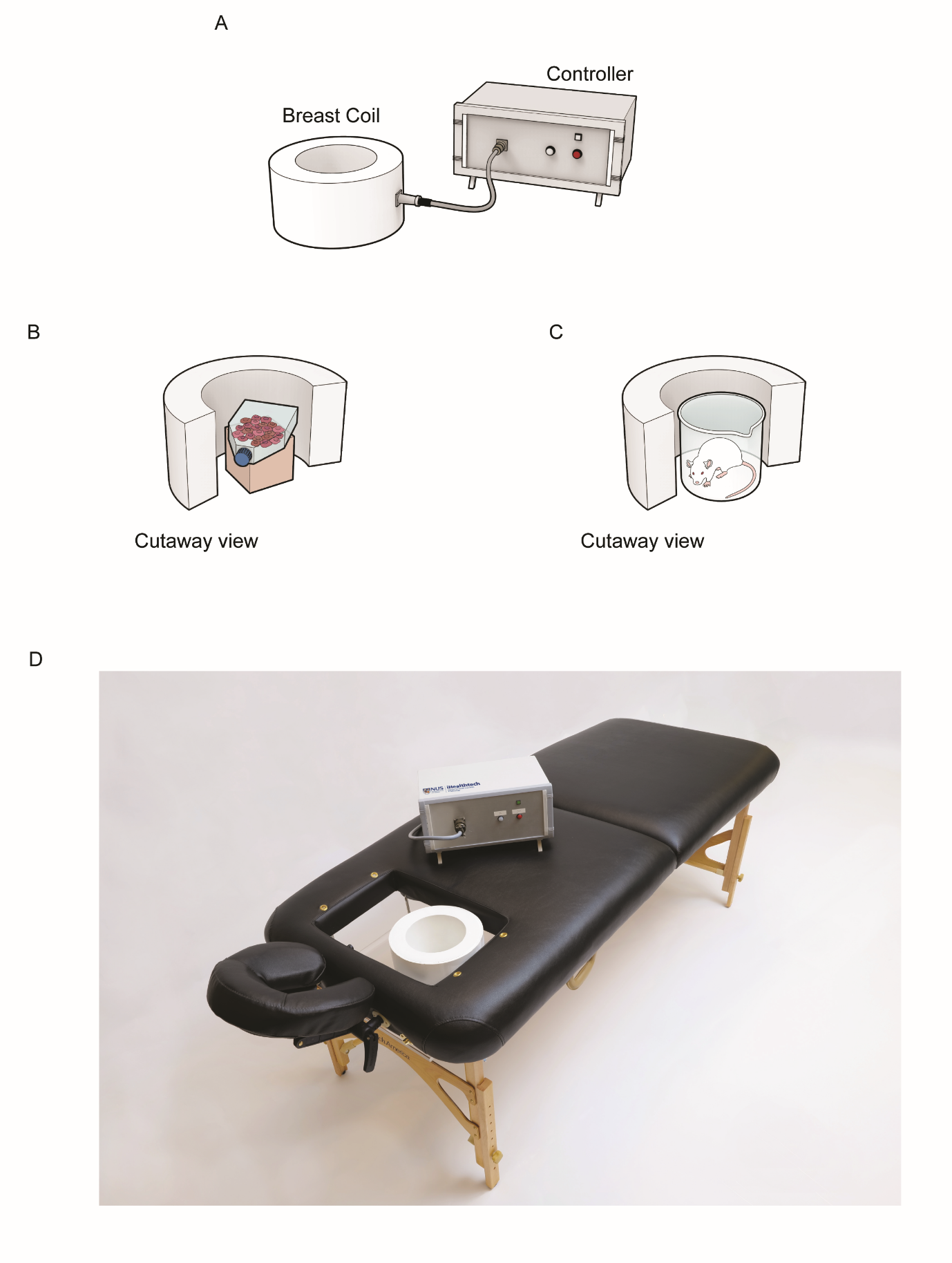
**

**Supplementary Figure 1. (A)** Illustration of human breast coil prototype. Efficacy validation method of the human breast coil prototype used in tissue culture **(B)** and mice **(C)** experiments. **(D)** Photograph of fully assembled breast coil system to be ultimately employed in human clinical trials. The lumen of the PEMF breast coil was roughly based on the dimensions of existing clinical MRI breast scanning coil devices that are designed to accommodate the human female breast.


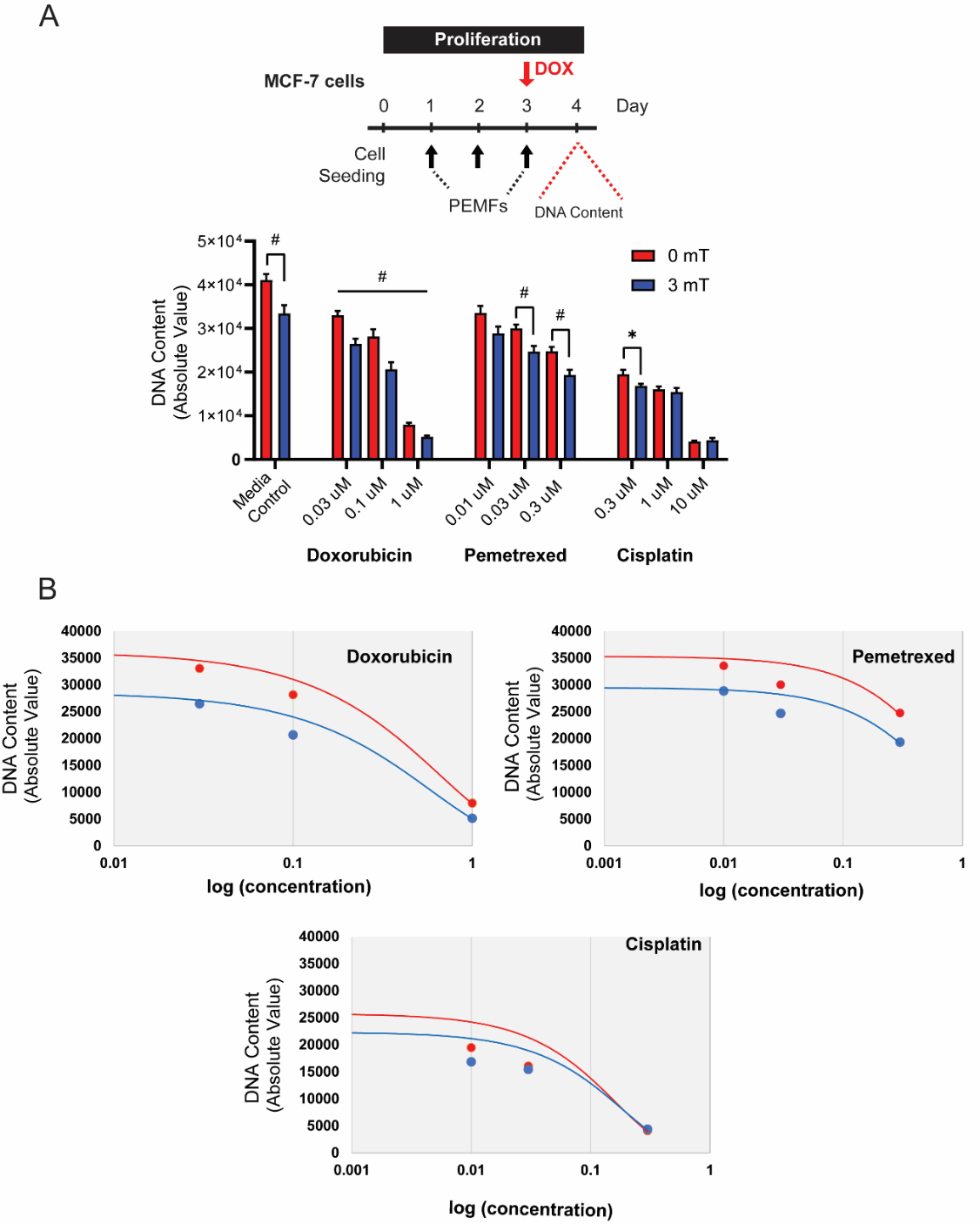


**Supplementary Figure 2. Chemo-sensitivity of MCF-7 cancer cells to pemetrexed and cisplatin in combination with PEMF in comparison to DOX.** **(A)** Cells were seeded in 96-well and exposed to 3 mT PEMF for 1 h per day for 3 days. Chemotherapeutic drugs were added (red arrow) on the final day of PEMF before the analysis of DNA content 24 h later. The corresponding bar charts show the absolute DNA content of cells in response to increasing doses of chemotherapeutic agents with or without PEMFs. **(B)** Representation of a dose**-**response curve of the same data in (A), treated with DOX, pemetrexed, and cisplatin. The X-axis represents log (concentration), and the y-axis represents the absolute intensity of DNA content. Statistical analysis was performed using multiple unpaired *t*-tests, comparing between 0 mT and 3 mT within each concentration. All experiments were from 3 to 5 independent experiments with **p* < 0.05 and ^#^*p* < 0.0001. The error bars are expressed as the standard error of the mean.


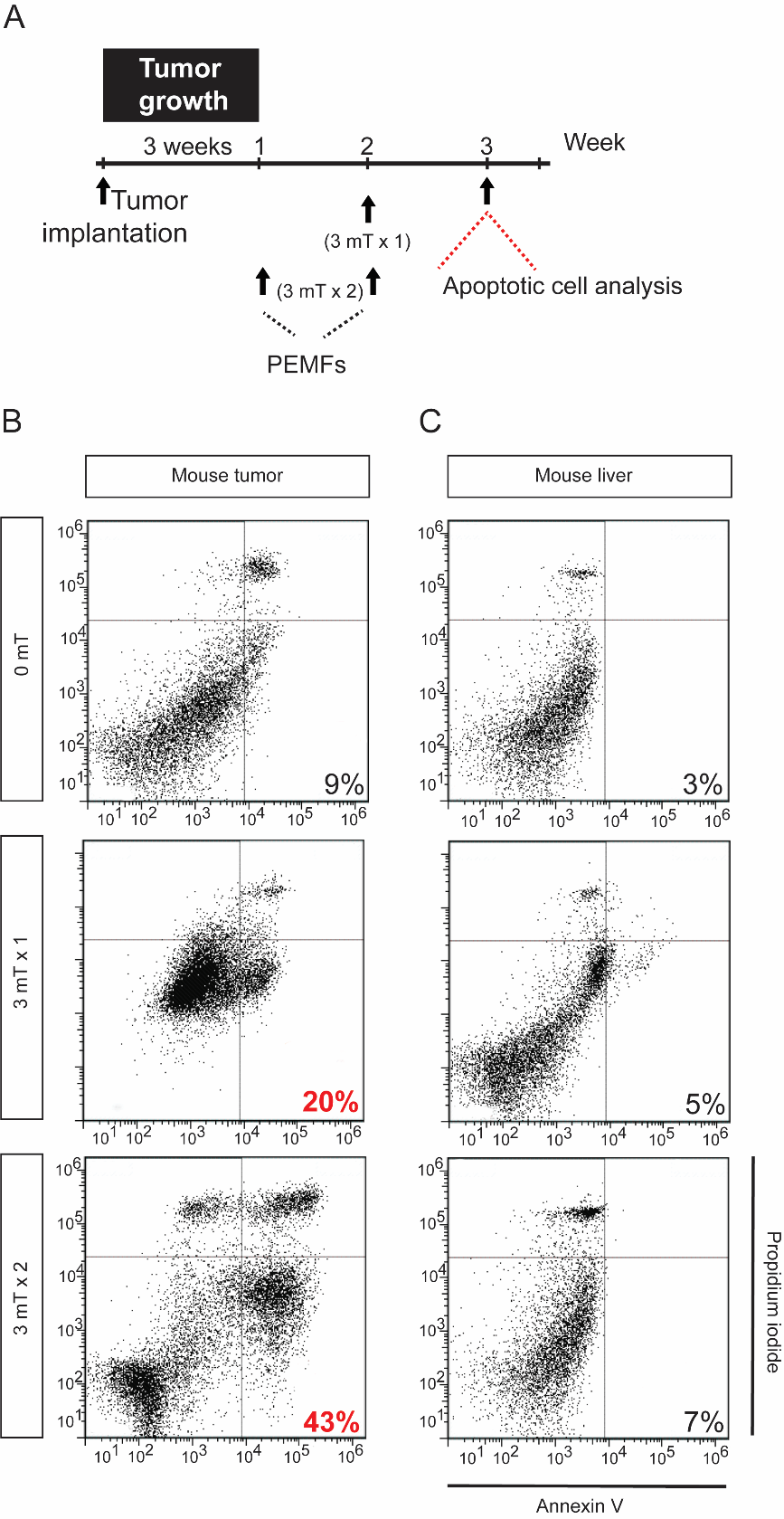


**Supplementary Figure 3. PEMFs inhibit MDA-MB-231 tumor growth without affecting liver *in vivo*. (A)** Schematic of PEMF exposure of NSG mice implanted with MDA-MB-231 tumors. Implanted tumors were allowed to grow for 3 weeks before the initiation of PEMF exposure once (3 mT x 1; 1 h, once a week) or twice (3 mT x 2; 1 h once a week for 2 weeks). Flow cytometric analysis was performed 1 week after the last PEMF exposure. Representative scatter dot-plots of **(B)** MDA-MB-231 xenografts and **(C)** mouse livers showing cell population of dissociated tumors sorted based on Annexin V and propidium iodide staining. The percentages repr
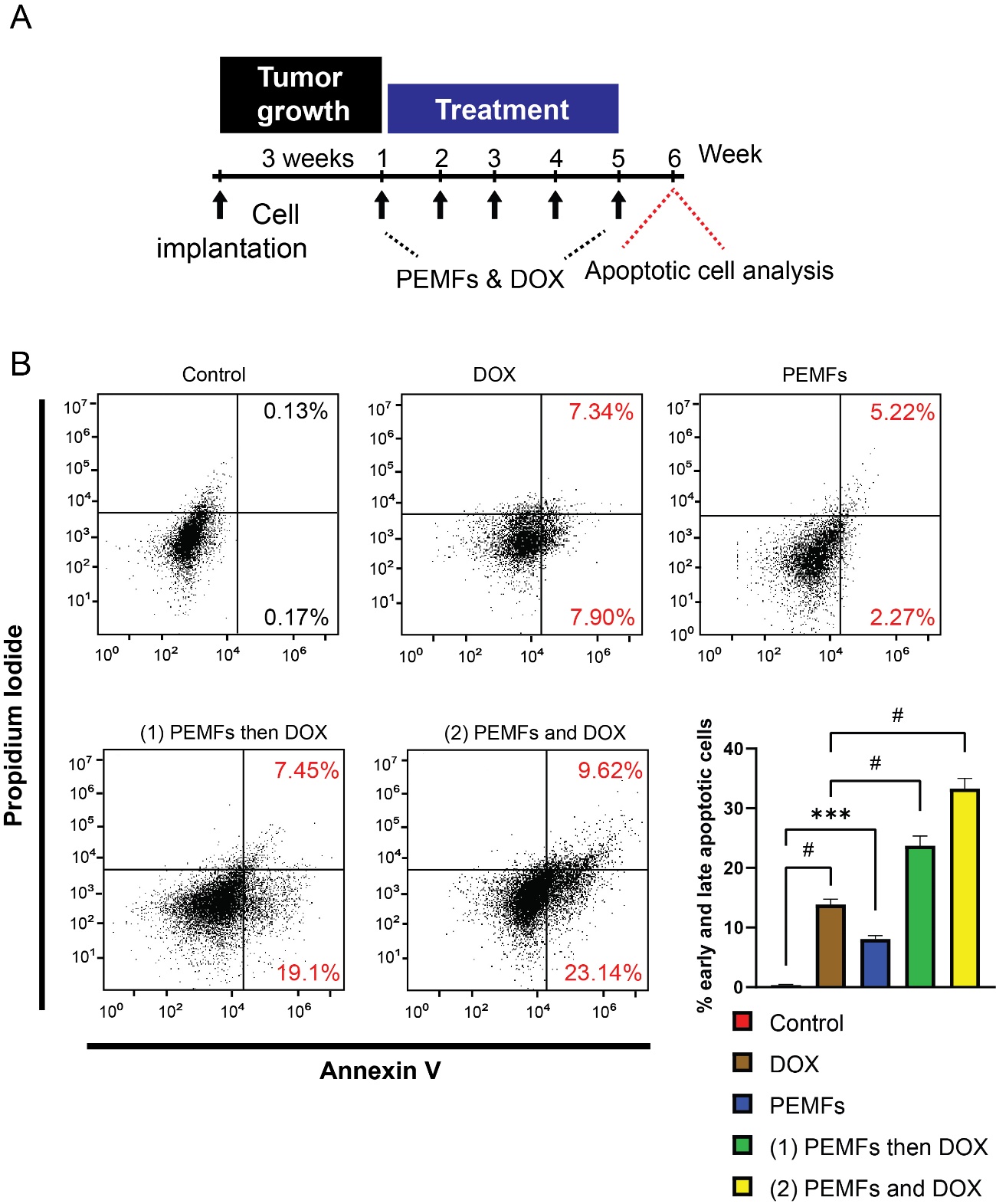
esent the total early and late apoptotic cells.

**Supplementary Figure 4. PEMFs synergize with doxorubicin to inhibit MCF-7 tumor growth *in vivo*. (A)** Schematic of weekly PEMF and DOX exposures on MCF-7 xenograft in mice. Implanted cells were allowed to grow for 3 weeks before the initiation of DOX and/or PEMF treatment. Apoptotic cell determination was performed at the end of the study. **(B)** Representative scatter dot-plots showing cell population of dissociated tumors sorted based on Annexin V and propidium iodide staining. Bar charts represent pooled data of early and late apoptotic cell percentages analyzed using flow cytometry. N = 6 mice, with ****p* < 0.001 and *^#^p* < 0.0001. The error bars are expressed as the standard error of the mean.


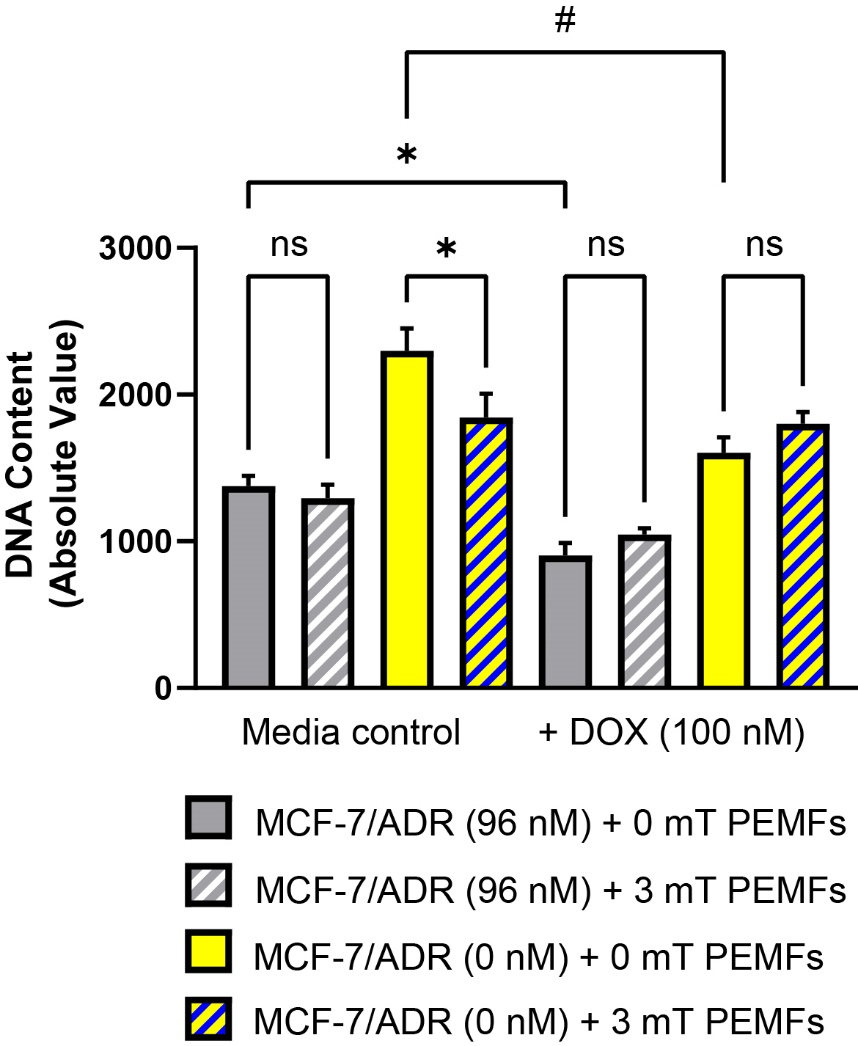


**Supplementary Figure 5**. **Recovery of PEMF cytotoxicity upon removal of selective pressure from DOX and reinstatement of TRPC1 expression.** Cell proliferation assay using Cyquant DNA content analysis on MCF-7/ADR cell lines, in combination with DOX (100 nM) and PEMF exposure. MCF-7/ADR (96 nM) cells were maintained in 96 nM DOX while MCF-7/ADR (0 nM) were serially passaged in the absence of DOX. Cells were exposed to 3 mT PEMFs for 1 h per day for 3 days. 100 nM DOX was administered 1 h before the final PEMF exposure. Cyquant analysis was performed 24 h after the final PEMF exposure. Statistical analysis was performed using One-Way ANOVA with Sidak’s multiple comparison test with **p* < 0.05 and ^#^*p* < 0.0001. Data presented were from 2 independent experiments with 6 technical replicates per experiment. The error bars are expressed as the standard error of the mean.
